# Bifunctional Hydrogels Containing the Laminin Motif IKVAV Promote Neurogenesis

**DOI:** 10.1016/j.stemcr.2017.09.002

**Published:** 2017-10-05

**Authors:** Aleeza Farrukh, Felipe Ortega, Wenqiang Fan, Nicolás Marichal, Julieta I. Paez, Benedikt Berninger, Aránzazu del Campo, Marcelo J. Salierno

**Affiliations:** 1 INM – Leibniz Institute for New Materials, Campus D2 2, 66123 Saarbrücken, Germany; 2 Biochemistry and Molecular Biology Department IV, Faculty of Veterinary Medicine, Complutense University, Madrid, Spain; 3 Institute of Neurochemistry (IUIN), 28040 Madrid, Spain; 4 Health Research Institute of the Hospital Clínico San Carlos (IdISSC), Madrid, Spain; 5 Institute of Physiological Chemistry, University Medical Center of the Johannes Gutenberg University Mainz, Hanns-Dieter-Hüsch-Weg 19, 55128 Mainz, Germany; 6 Focus Program Translational Neuroscience, Johannes Gutenberg University Mainz, 55131 Mainz, Germany; 7 Saarland University, Campus Saarbrücken D2 2, 66123 Saarbrücken, Germany

**Keywords:** biomaterials, bioengineering, hydrogels, neural stem cells, cell differentiation, laminin, IKVAV, polylysine, β_1_-integrin, neurogenesis

## Abstract

Engineering of biomaterials with specific biological properties has gained momentum as a means to control stem cell behavior. Here, we address the effect of bifunctionalized hydrogels comprising polylysine (PL) and a 19-mer peptide containing the laminin motif IKVAV (IKVAV) on embryonic and adult neuronal progenitor cells under different stiffness regimes. Neuronal differentiation of embryonic and adult neural progenitors was accelerated by adjusting the gel stiffness to 2 kPa and 20 kPa, respectively. While gels containing IKVAV or PL alone failed to support long-term cell adhesion, in bifunctional gels, IKVAV synergized with PL to promote differentiation and formation of focal adhesions containing β_1_-integrin in embryonic cortical neurons. Furthermore, in adult neural stem cell culture, bifunctionalized gels promoted neurogenesis via the expansion of neurogenic clones. These data highlight the potential of synthetic matrices to steer stem and progenitor cell behavior via defined mechano-adhesive properties.

## Highlights

• Coupling of polylysine and the laminin peptide IKVAV renders hydrogels bifunctional
• Bifunctional hydrogels promote neurogenesis from neural stem cells (NSCs)
• Hydrogel stiffness differentially affects neurogenesis from embryonic and adult NSCs
• Surface bifunctionality promotes formation of focal adhesions containing β_1_-integrin

## Teaser

In this article, Farrukh and colleagues show that bifunctionalization of hydrogel substrates with polylysine and a 19-mer peptide containing the laminin motif IKVAV promotes neurogenesis from embryonic neuroblasts and adult neural stem cells. Neurogenesis and neurite outgrowth can be further optimized by adjusting gel stiffness in a cell-type-specific manner.

## Introduction

There is a surge of interest in designing hydrogels endowed with specific mechanical and chemical properties for steering cell behavior, including cell-fate decision and plasticity as well as organoid formation (Engler et al., 2006, Floren et al., 2016, Gjorevski et al., 2016, Her et al., 2013, Wen et al., 2014). Previously, we have studied lineage progression of neural stem cells (NSCs) from the adult subependymal zone (SEZ) using hard surfaces coated with polylysine (Costa et al., 2011, Ortega et al., 2011), leaving the question open whether the observed patterns of cell division and terminal differentiation within single clones can be altered by modifying the mechanical and chemical properties of the substrate. In fact, there is ample evidence that stem cell behavior is under strong influence of the molecular milieu provided by their niche (Theocharidis et al., 2014). One component shown to regulate the activated state of adult SEZ NSCs is the basement membrane glycoprotein laminin (Kokovay et al., 2010). Tashiro and colleagues identified a 19-mer peptide containing the pentapeptide IKVAV within the laminin α1 chain as one of the principle sites in laminin to regulate cellular behavior (Sephel et al., 1989, Tashiro et al., 1989). IKVAV was found to enhance viability and maturation of neurons by binding to the β_1_-integrin subunit (Agius et al., 1996, Li et al., 2014, Sur et al., 2012, Tashiro et al., 1989), rendering it a promising candidate for use in neuronal growth-stimulating materials.

Previously, we engineered bifunctionalized polyacrylamide gels (PA) allowing orthogonal coupling of thiol- and amine-containing ligands (Farrukh et al., 2016). Subsequently, we used this bifunctionalized PA to couple polylysine (via its amine group) and IKVAV (via its thiol group) independently to the substrate and studied its effect on neuronal survival and neurite outgrowth (Farrukh et al., 2017). In the present study, we aimed to optimize the mechanical properties of these bifunctionalized gels for culturing embryonic and adult neural progenitors by applying distinct stiffness regimes. Our work provides a rationale for further study on the consequences of culturing neural progenitors in microenvironments of enriched chemical functionality and mechanical properties.

## Results

### Neurite Outgrowth of E14.5 Cortical Neurons Is Enhanced on 2 kPA IKVAV/PL Bifunctionalized Gels

**Figure 1:**
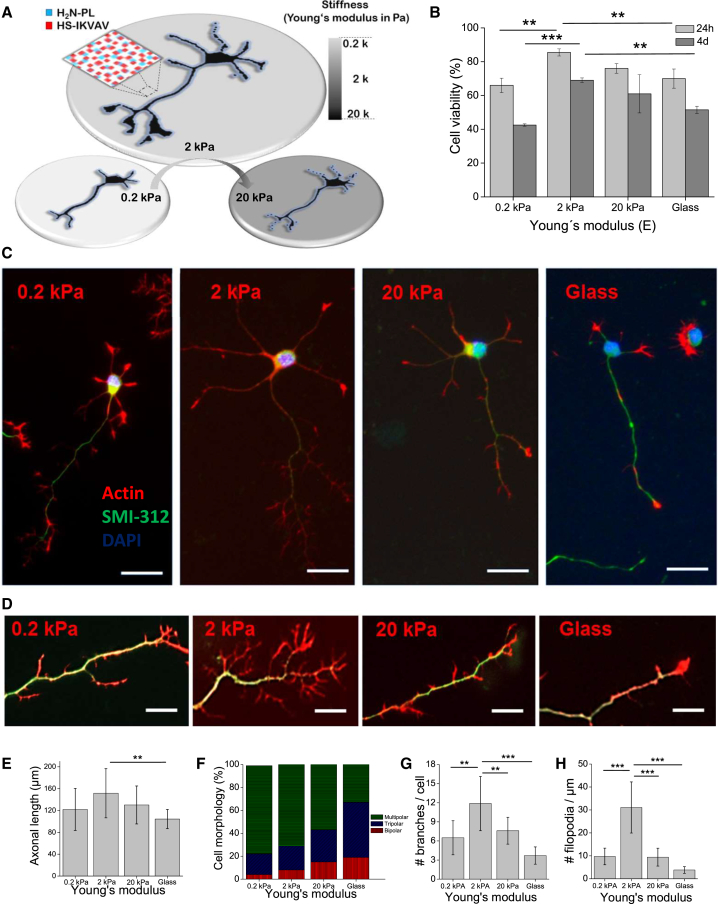
Development of Cortical Progenitors on Bifunctionalized IKVAV/PL Substrates at Different Stiffnesses (A) Substrate platform based on PA gels containing two different biomolecules covalently immobilized with orthogonal coupling using thiols for IKVAV (red squares) and amines for PL (blue squares). In addition, hydrogels were adjusted at different Young's modulus E (kPa) in the range of soft tissue rigidities (i.e., 0.2, 2, and 20 kPa). (B) Viability of cortical progenitor cells after 24 hr and 4 DIV cultured on bifunctionalized substrates at different gel stiffnesses. 2 kPa gels significantly improved neuronal viability (mean ± SD, ANOVA, Tukey-Kramer post hoc test ^∗∗^p < 0.01, ^∗∗∗^p < 0.001). (C) Representative pictures of a single cortical progenitor after 4 DIV cultured on substrates at different stiffness (scale bar, 20 μm). (D) The formation of actin filopodia (phalloidin reaction, in red) was appreciably incremented on 2 kPa IKVAV/PL gels, in particular along the axon (SMI-312 immunoreactivity, in green). Scale bar, 10 μm. (E) Average axon length was significantly increased in 2 kPa bifunctionalized gels compared with PL coated on glass. (F) Distribution of number of primary neurites on cortical progenitor cells. The amount of neurites displayed a reverse correlation with the degree of gel stiffness. (G and H) Average number of secondary branches (G) and dendritic filopodia (H) show a significantly increase on 2 kPa gel stiffness. Contrast were determined a *posteriori* against bifunctionalized 2 kPa gels which displayed the optimal mechanical conditions (n = 3 independent experiments, 20–25 fields of view). Data in (E, G, and H) are represented as means ± SD, Tukey post hoc test (^∗∗^p < 0.01, ^∗∗∗^p < 0.001).

We first evaluated the effect of bifunctionalized IKVAV/PL gels at different gel stiffness to determine the optimal mechanical conditions for neuronal differentiation of E14 embryonic cortical progenitors. Cells were seeded on substrates within the range of soft tissue stiffness (0.2, 2, and 20 kPa) (Discher et al., 2005) as depicted in Figure 1A. Viability of cortical progenitors was significantly improved on 2 kPa IKVAV/PL gels compared with the other stiffness regimes (Figure 1B). Likewise, on day 4 of culture, neurite growth was enhanced on 2 kPa IKVAV/PL gels (Figure 1C), with an appreciable increment in actin filopodia (Figure 1D). Also, average axon length (identified by SMI-312 immunoreactivity, see Experimental Procedures) was significantly increased in 2 kPa IKVAV/PL gels compared with PL coating on glass (Figure 1E). Noticeably, the number of primary neurites displayed a reverse correlation with the degree of stiffness, with 0.2 kPA being most favorable for the protrusion of multiple primary processes (Figure 1F). However, the number of dendritic filopodia and secondary branches exhibited peak growth on 2 kPa gels compared with other levels of stiffness (Figures 1G and 1H). In fact, the number of dendritic filopodia was found to be the morphological feature that was most sensitive to mechanical changes. Further experiments demonstrated that neurite outgrowth was similar when the IKVAV containing 19mer was replaced by the complete laminin protein (Figures S1B–S1E). In contrast, substitution of IKVAV with another laminin motif cRGDfC (Yamada, 1991) on 2 kPa substrates showed no improvement of neurite outgrowth (Figures S1B–S1E), highlighting the specificity of the effect induced by the IKVAV motif.

### Bifunctionalized IKVAV/PL Gels Accelerate Maturation and Increase the Adhesiveness of Cortical Progenitors

**Figure 2:**
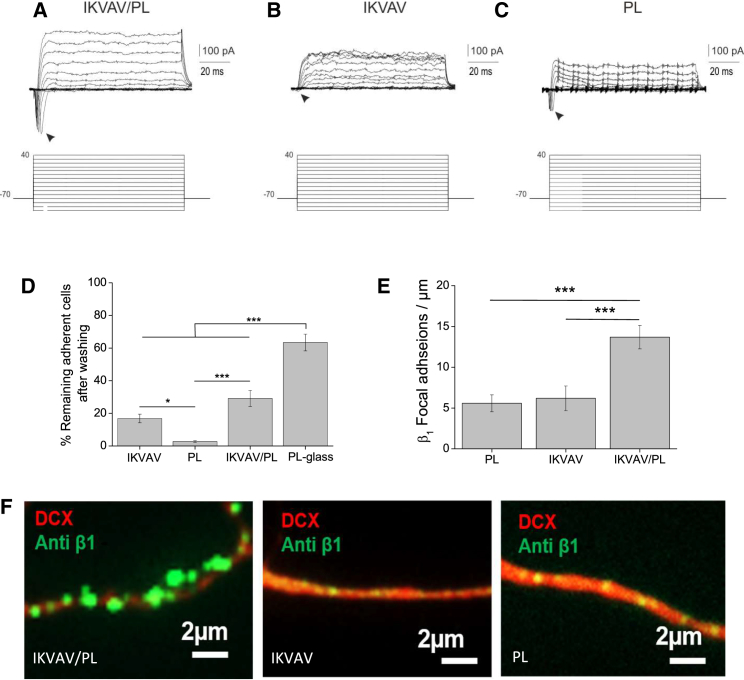
2 kPa IKVAV/PL Gels Accelerate Maturation and Enhance Adhesiveness of Cortical Neurons (A–C) Patch-clamp recording in a representative cell under different substrate conditions: IKVAV/PL (A), IKVAV (B), or PL (C). Graphics show leak-subtracted currents in response to a series of voltage steps. Arrowheads highlight voltage-dependent inward currents. (D) Cell-substrate adhesion assay showed that cortical neurons significantly increase the adherence on IKVAV/PL gels after 48 hr. The average cell number for each condition before and after washing is shown in Figure S2A. (E) Average focal adhesions containing β_1_-integrin in neurons after 5 DIV. Substrates with IKVAV/PL bifunctionalization significantly increase β_1_-integrin in comparison with single-gel coupling of IKVAV or PL. (F) Focal adhesions containing β_1_-integrin (green) along the neurites (DCX, red) substantially increase on IKVAV/PL bifunctionalized substrates (see also Figure S2B). Data in (D) and (E) are represented as means ± SD, n = 3 independent experiments, 20–25 fields of view, Tukey post hoc test (^∗^p < 0.05, ^∗∗∗^p < 0.001).

We next addressed whether the enhanced morphological differentiation of early cortical neurons on 2 kPa IKVAV/PL gels correlated with increased physiological maturation. Using patch-clamp recording, we found that neurons on 2 kPA IKVAV/PL gels displayed well-developed fast voltage-gated inward currents (Figure 2A) typical of Na^+^ channel activation (*I*_Na_ maximum peak amplitude −278.4 ± 69 pA, n = 8 cells) similar to young neurons in acute or cultured embryonic cortical slices (Noctor et al., 2004). Remarkably, neuronal cultures grown on 2 kPa gels functionalized only with a single molecule, i.e., either IKVAV or PL alone (Figures 2B and 2C, respectively), displayed inward voltage-gated currents with noticeably lower amplitudes (*I*_Na_ maximum peak amplitude −57.6 ± 34.3 pA, n = 5 cells, mean ± SEM, p < 0.05, Mann-Whitney U test). These data suggest a synergism of the IKVAV and PL functional groups to promote maturation of the intrinsic properties of cortical neurons.

### IKVAV Synergized with PL to Increase Adhesiveness and Focal Adhesions Containing β1-Integrin in Cortical Progenitors

Finally, we performed a cell-substrate adhesion assay (Humphries, 2001) to explore the adhesive properties of the bifunctionalized gels compared with single IKVAV or PL coupling. The cell ratio (i.e., cells after/cells before the washing procedure, see Figure S2A) declined significantly on all gel surfaces compared with PL-coated glass slides (Figure 2D). However, gels containing the IKVAV motif significantly increased cell adhesion compared with single PL coupling on gels, highlighting the adhesive effect of the short laminin sequence. In addition, as IKVAV is known to directly bind to the transmembrane receptor β_1_-integrin (Agius et al., 1996, Nomizu et al., 1995), we quantified focal adhesions in cortical neurons 48 hr after plating. Immunocytochemistry revealed a significant increase in focal adhesions containing β_1_-integrin on bifunctional IKVAV/PL substrates compared with monofunctional gels (Figure 2E). Intriguingly, β_1_-integrin immunoreactivity appeared to be denser and expanded along the neurites extended on IKVAV/PL substrates (Figures 2F and S2B), consistent with the increased neurite outgrowth observed above.

### IKVAV/PL Bifunctionalized Gels Enhance Adult NSC Symmetric Neurogenic Divisions and Promote Overall Neurogenesis

**Figure 3:**
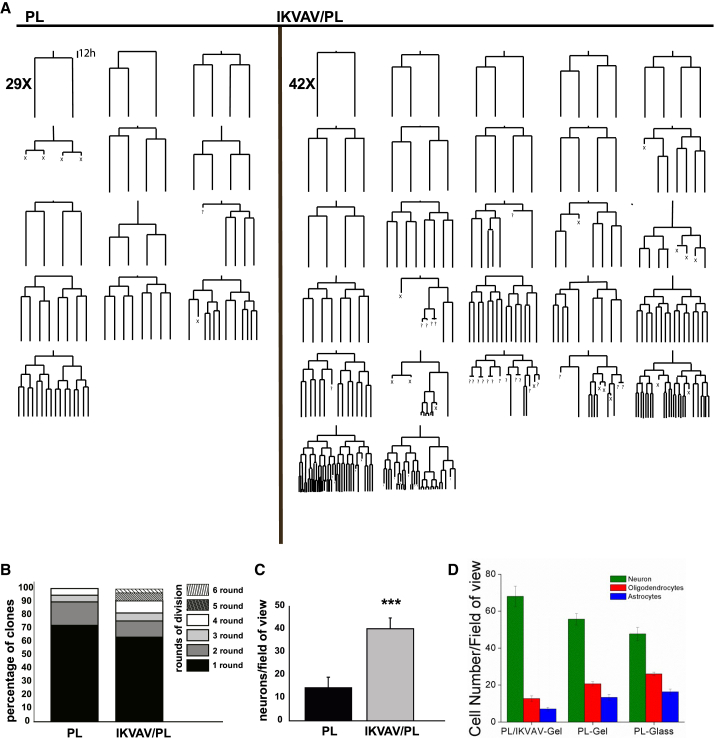
Neurogenic Development of aNSCs Clones: Comparison of IKVAV/PL versus PL Gel Platforms at 20 kPa Stiffness (A) Lineage trees representing the clonal evolution of single aNSCs *in vitro*. The figure compares the trees tracked under PL gels (left) versus IKVAV/PL gels (right) at 20 kPa stiffness. Bifunctional gels produced a higher number of clones. Tree reconstructions showed mostly symmetric distribution with more rounds of divisions on IKVAV/PL gels. (B) Histogram showing the fraction of clones per round of division. IKVAV/PL gels presented 6% of clones containing 5 rounds of division and 3% of the symmetric neurogenic clones with an unusual 6 rounds of division. (C) Number of mature neurons per field of view after 5 DIV. t test (^∗∗∗^p < 0.001). (D) Average number for a particular cell type (neurons, astrocytes, and oligodendrocytes) per field of view on 20 kPa IKVAV/PL gels, 20 kPa PL gels, or PL coated on glass. Significant differences for each cell type between conditions are shown in Figure S3B. Data are represented as means ± SEM, n = 3 independent experiments.

Next, we investigated the effect of IKVAV/PL gels on adult neural stem cells (aNSCs) derived from the adult SEZ. In notable contrast to the results obtained with E14.5 cortical progenitors, aNSCs cultures showed reduced adherence and poorer survival on 0.2 and 2 kPa IKVAV/PL gels (data not shown). In further contrast, 20 kPa IKVAV/PL gels supported substantial cell survival of aNSC, allowing lineage progression toward neurogenesis. Therefore, we examined the generation of lineage trees (Figures 3A and S3A for a description of the lineage trees) via single-cell tracking of aNSCs on 20 kPa bifunctionalized IKVAV/PL gels and monofunctionalized 20 kPa PL gels (monofunctionalized 20 kPa IKVAV gels also failed to support substantial cell survival). Interestingly, aNSCs grown on bifunctional IKVAV/PL gels displayed a higher percentage of symmetric lineage trees with more rounds of divisions (Figure 3B) leading to ∼3 times increase in the number of neurons in comparison with PL gels (Figure 3C). Intriguingly, the effect of IKVAV ligand on promoting cell neurogenesis was further supported by a significant decrease of glial lineages in absolute numbers (i.e., astrocytes and oligodendrocytes) on IKVAV/PL gels in comparison with PL gels or PL coated on glass (Figures 3D and S3B).

### Maturation and Neurite Outgrowth of Adult NSCs Are Accelerated on 20 kPA IKVAV/PL Bifunctionalized Gels

**Figure 4:**
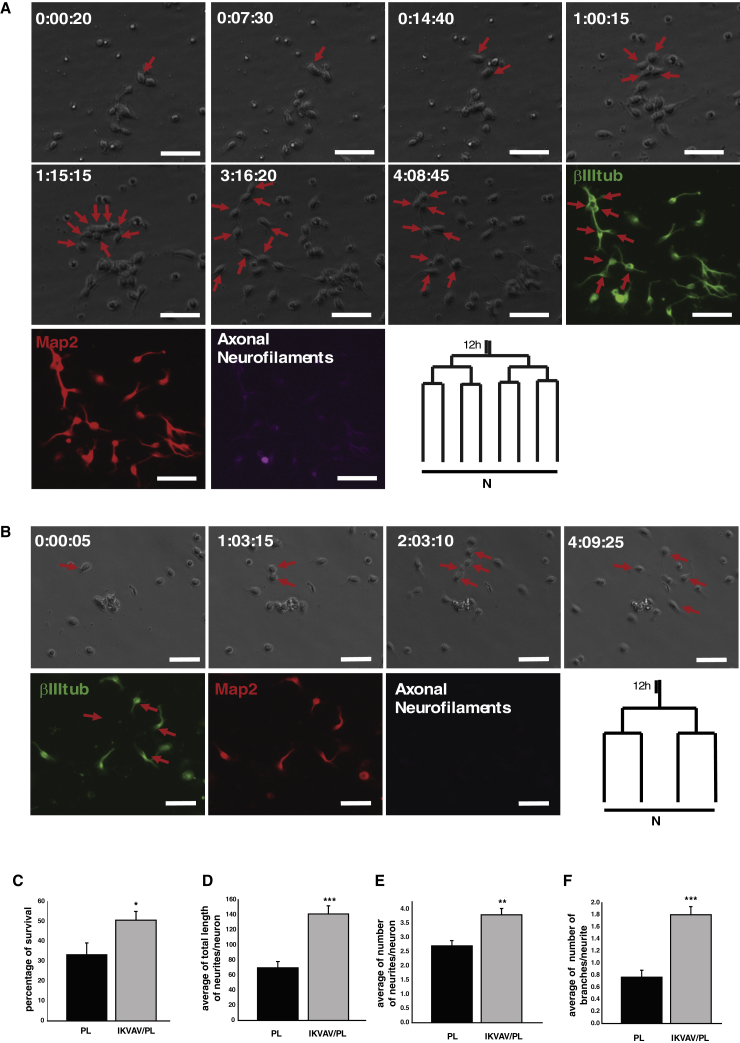
IKVAV/PL Gels Increase aNSCs Survival, Maturation, and Neurite Outgrowth (A and B) Pictures showing the time lapse of a representative aNSC clone (day:hour:minute) on 20 kPa IKVAV/PL gels (A) or PL gels (B). Red arrows indicate the clonal expansion that was represented in a lineage tree for each case. The last three pictures show the post-imaging staining for neuronal markers βIII tub (green), Map2 (red), and the axonal neurofilament marker SMI-312 (purple). (C–F) Percentage of viable cells after 5 DIV. Analysis of neurite outgrowth: average length of neurites per neuron (D), average number of neurites per neuron (E), and average number of secondary branches per neurite (F). Data are represented as means ± SEM, t test (^∗^p < 0.05, ^∗∗^p < 0.01, ^∗∗∗^p < 0.001), n = 3 independent experiments. Scale bar, 50 μm.

Moreover, single-cell tracking showed that besides the increase in Map2-positive neurons on 20 kPa IKVAV/PL gels at the clonal level, these neurons also expressed axon-specific neurofilament identified by SMI-312 immunoreactivity (Figure 4A), while neurofilament expression was not detected at the same stage in neuronal clones grown on 20 kPA PL gels (Figure 4B). This suggests a higher degree of maturation of aNSC-derived neurons on bifunctionalized IKVAV/PL gels. In addition, neuronal survival was improved resulting in a higher percentage of viable progressing neurons (∼50%) toward maturation (Figure 4C). In agreement with our data in cultures of cortical progenitors, aNSCs exhibited a significant increase in neurite outgrowth on IKVAV/PL gels compared with PL gels after 5 days in vitro (DIV) (e.g., neurite length, number, and branches) (Figures 4D–4F).

## Discussion

Here, we demonstrate that PA gels containing IKVAV and PL are a valuable design to enhance neurogenesis and promote neurite outgrowth. Furthermore, we provide evidence for the importance of adjusting gel elasticity in a cell-type-specific manner. Notably, embryonic cortical progenitors and adult NSCs were optimally cultured on gels with different matrix elasticity (2 kPA and 20 kPA, respectively). Beyond the adjustment of the substrate stiffness, functionalization with the IKVAV motif appeared as an important pro-neurogenic factor, in agreement with recent studies which employed this motif in biomaterials to improve cultures of dorsal root ganglial cells, induced pluripotent stem cells-neural progenitor cells, and hNSCs (Berns et al., 2014, Lam et al., 2015, Li et al., 2014). Yet, bifunctionalization by orthogonally coupling with IKVAV and PL worked synergistically to boost neurogenic IKVAV properties while also promoting cell adhesion. IKVAV/PL synergism was found to increment focal adhesions containing β_1_-integrin in embryonic cortical neurons, particularly on their neurites, providing a mechanistic explanation for the observed increase in overall cell adhesiveness and enhanced neurite outgrowth. This is in line with previous studies showing that β_1_-integrin promotes neurogenesis and enhances proliferation upon expression of a constitutively active β_1_-integrin in the embryonic chick mesencephalon (Long et al., 2016), and that ablation of β1-integrin leads to reduced neurogenesis from hippocampal NSCs (Brooker et al., 2016).

IKVAV/PL gels modulated lineage progression of the SEZ-derived aNSCs (Ortega et al., 2011, Ponti et al., 2013) by increasing the rounds of amplifying divisions. This is consistent with previous results showing that IKVAV increases proliferation of cultured human NSCs (Li et al., 2014). The IKVAV peptide is not only important for regulation of NSC proliferation but is also directly connected to the promotion of neuronal maturation and survival in hNSCs, murine cerebellar granule neurons, and Purkinje neurons (Li et al., 2014, Sur et al., 2012, Sur et al., 2014). In addition, our study showed reduced gliogenesis from adult NSCs cultured on IKVAV/PL gels. This is in line with a recent study demonstrating that the injection of the IKVAV epitope into the ependymal region induces high levels of β_1_-integrin in ependymal stem cells *in vivo*, resulting in a reduction of astrogliogenesis (Pan et al., 2014). Previously, pioneering work showed that self-assembly of nanotubes from amphiphilic peptides containing IKVAV inhibited glial differentiation in NSC cultures (Silva et al., 2004); yet, this differentiation suppressive effect was not observed when applying the IKVAV peptide in solution in the absence of the charges provided by the remainder of the amphiphilic peptide. In a similar manner, only bifunctionalization of IKVAV with PL in PA gels succeeded in significantly improving neuronal maturation and survival in comparison with monofunctionalized gels, stressing that IKVAV mimetic peptide should be inserted in a favorable environment in order to boost neurogenesis.

In summary, the use of bifunctionalized IKVAV/PL gels is a novel strategy to enhance neurogenesis *in vitro*. Moreover, optimization of the substrate elasticity enhanced the biological action of IKVAV. Thus, PA gels represent platforms with highly tunable mechanical properties (Kandow et al., 2007) that cover the entire range of elastic modulus encountered in animal soft tissues (Yeung et al., 2005). The platform presented here allows modulation of mechanical properties while defining adhesive properties via orthogonal coupling of IKVAV and PL to govern cellular behavior. Future developments of this strategy may allow improved control of organoid development as has been recently proved in the case of intestinal stem cells (Gjorevski et al., 2016). Here, we demonstrate that cell-surface interactions together with appropriate mechanical properties can increase cell survival, direct cell fate, and boost differentiation, highlighting the usefulness of engineered substrates for controlling stem cell cultures.

## Experimental Procedures

### Embryonic and Adult Cortical Progenitor Isolation and Culture

Cerebral cortex from E14.5 of C57BL/6 mice was digested in 0.5% trypsin EDTA (GIBCO) for 15 min at 37°C. Trypsin was inactivated with DMEM (GIBCO) and 10% fetal bovine serum (Hyclone) and gently triturated with a 5 mL disposable pipette to get a single cell. After centrifuging at 1,000 rpm for 5 min, cell pellets were re-suspended with 1 mL of differentiating medium (DMEM +2% B27 supplement from Invitrogen). Substrates were placed in a 24-well plate seeding 5 × 10^4^ cells in each well. For adult NSC culture, SEZ cultures were prepared from the lateral wall of the adult SEZ of young adult (8–12 weeks) C57/BL6 mice as previously described (Ortega et al., 2011).

### Live-Cell Imaging

A cell observer (Zeiss) and a Nikon TE-2000 were used for time lapse studies at a constant temperature of 37°C and 8% CO_2_. Images were acquired every 5 min using a 20× PH objective (Zeiss or Nikon), an AxioCamHRm/ZYLA from ANDOR cameras, and Zeiss AxioVision 4.7/NIS-elements from Nikon software.

### Single-Cell Clonal Trees

Trees were reconstructed from single-cell tracking using a self-written computer program (tTt) (Hilsenbeck et al., 2016). The identity of the progeny was determined by post-imaging ICC as previously described (Ortega et al., 2011).

### Cell Viability Analysis

Cell viability (%) = cells at t_(24 hr or 4 days)_/cells at t_0_. Cell density was obtained from time-lapse videos starting 2 hr after seeding (t_0_).

### Immunofluorescence and Focal Adhesion Quantification

Cells were fixed with prewarmed 4% (v/v) paraformaldehyde for 15 min, washed with PBS, permeabilized using 0.5% (v/v) Triton X-100 in PBS for 15 min and washed with PBS. Cells were stained with the appropriate primary antibodies (see Supplemental Information) prepared in 20% (v/v) horse serum (Gibco) for either 2 hr at room temperature or overnight at +4°C, washed with PBS and incubated with secondary antibodies prepared in 30% horse serum and PBS for at least 60 min. The nucleus was stained using DAPI. Individual focal adhesions were identified using anti-β_1_-integrin antibody (Abcam, ab95623) and counted using the analyze particles built-in function of ImageJ.

### Electrophysiological Recordings

Patch-clamp whole-cell recordings were made using an Axopatch 200B (Molecular Devices) amplifier, which allowed voltage-clamp measurements (see Patch-clamp recording in Supplemental Information for more detail).

### Hydrogel Preparation

Gel stiffness was prepared by following a previously reported protocol (Farrukh et al., 2016, Farrukh et al., 2017) by free-radical copolymerization of methylsulfone acrylate, acrylamide, and acrylic acid with varying amount of N,N′-methylene-bis-acrylamide crosslinker to set PA gels at 0.2, 2, and 20 kPa (see Table S1). The physical properties of the gels obtained coincide with the literature (Farrukh et al., 2016, Farrukh et al., 2017, Zouani et al., 2013). Both binding strategies are stable under physiological conditions and also in the presence of cell culture medium, imparting uniform surface distribution of ligand.

### Statistical Analysis

All experiments comprised at least three independent experimental batches. Data are expressed as means ± SD. Statistical analysis for parametric results used the t test, ANOVA with a post hoc Tukey or Mann-Whitney U test. Statistical significance was determined between groups from p < 0.05.

## Author Contributions

A.F. prepared all substrates, designed and conducted experiments, and analyzed data. F.O. designed and conducted experiments and analyzed the aNSC data. W.F. conducted experiments, analyzed data, and performed cortical progenitor cultures. N.M. conducted the electrophysiological experiments. J.I.P., A.C., and B.B. provided critical revisions to the manuscript. M.J.S. designed experiments, analyzed data, and wrote the manuscript. All authors approved the final manuscript.

## Supplemental Information

Document S1. Supplemental Experimental Procedures, Figures S1–S3, and Table S1

Document S2. Article plus Supplemental Information
