# Supplementary material for: Bifunctional Hydrogels Containing the Laminin Motif IKVAV Promote Neurogenesis
Source: Stem Cell Reports. 2017 Oct 5;9(5):1432–40. doi: 10.1016/j.stemcr.2017.09.002 (PMC5829305; doi:10.1016/j.stemcr.2017.09.002)
Supplement: Document S1. Supplemental Experimental Procedures, Figures S1–S3, and Table S1 [file mmc1.pdf]

Stem Cell Reports, Volume *9*

Supplemental Information

Bifunctional Hydrogels Containing the Laminin Motif IKVAV Promote Neurogenesis

Aleeza Farrukh, Felipe Ortega, Wenqiang Fan, Nicolás Marichal, Julieta I. Paez, Benedikt Berninger, Aránzazu del Campo, and Marcelo J. Salierno

Supplemental figures

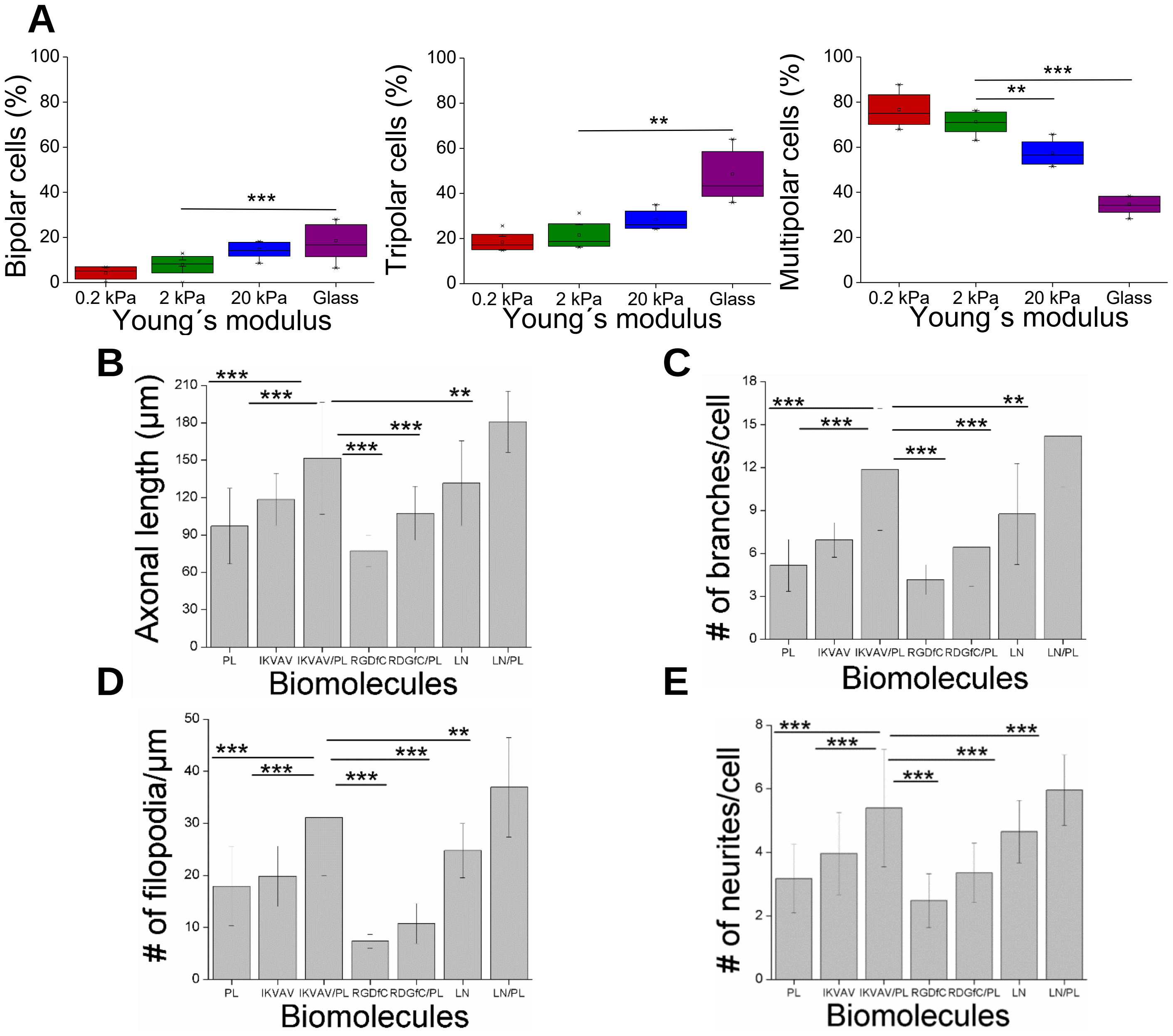

Figure SI1 related to figure 1. a) Quantification of average cell main processes depicting morphological changes in cortical progenitor population on IKVAV/PL-gels at different stiffness after 5 DIV: 2 neurites (bipolar), 3 neurites (tripolar) and >4 neurites (multipolar) (n = 54-60 cells, IQR: 25-75 percentile; whiskers: 1.5 x IQR, Kruskal-Wallis, Dunn's test ** p <0.01, *** p <0.001). Rounded cells were not included and there were no significant number of unipolar cells to be considered. Comparative analysis of matrix derived peptide sequences on eCPCs. 2 kPa-gels were functionalized with laminin and laminin-derived recognition sequences (cRGDfC, IKVAV) with or without PL. After 5 DIV morphological changes were compared: axonal length **(b)**, number of branches **(c)**, number of filopodia **(d)** and number of neurites **(e)**. For b to e data are represented as mean ± SD (n = 3 independent experiments), ANOVA, Tukey-Kramer post hoc test ** p <0.01, *** p <0.001.

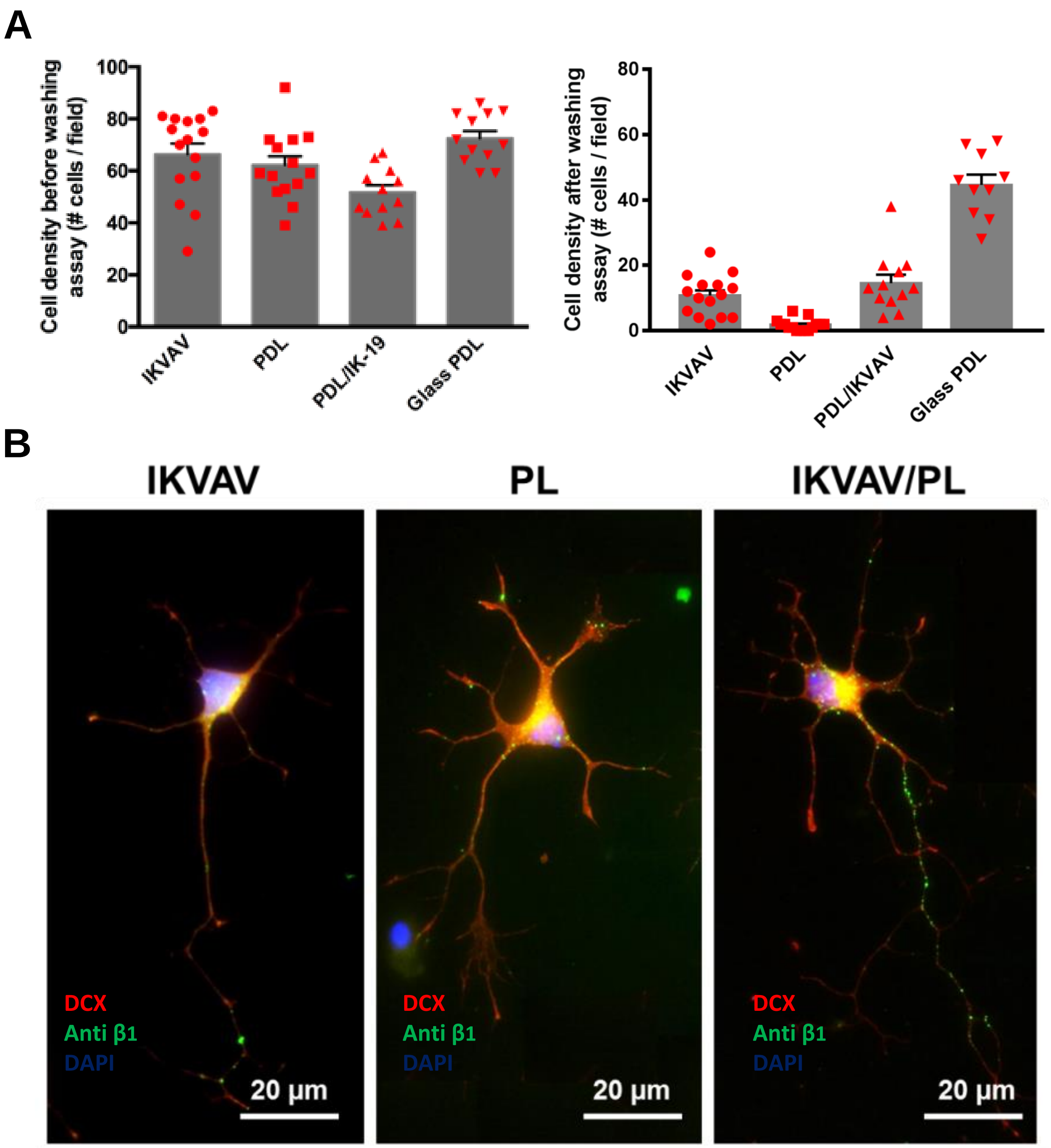

Figure SI2 related to figure 2. a) Average cell density before and after performing cell-substrate adhesion assay on each conditions. There were no significant differences between treatments before the test. Data are represented as mean ± SD (n = 3 independent experiments). **b)** Representative images of neurons after 5 DIV, stained with anti-β1 (green), DAPI (blue) and DCX (red) showing an increased density of focal adhesion containing β1 integrins in cells on IKVAV/PL-gels.

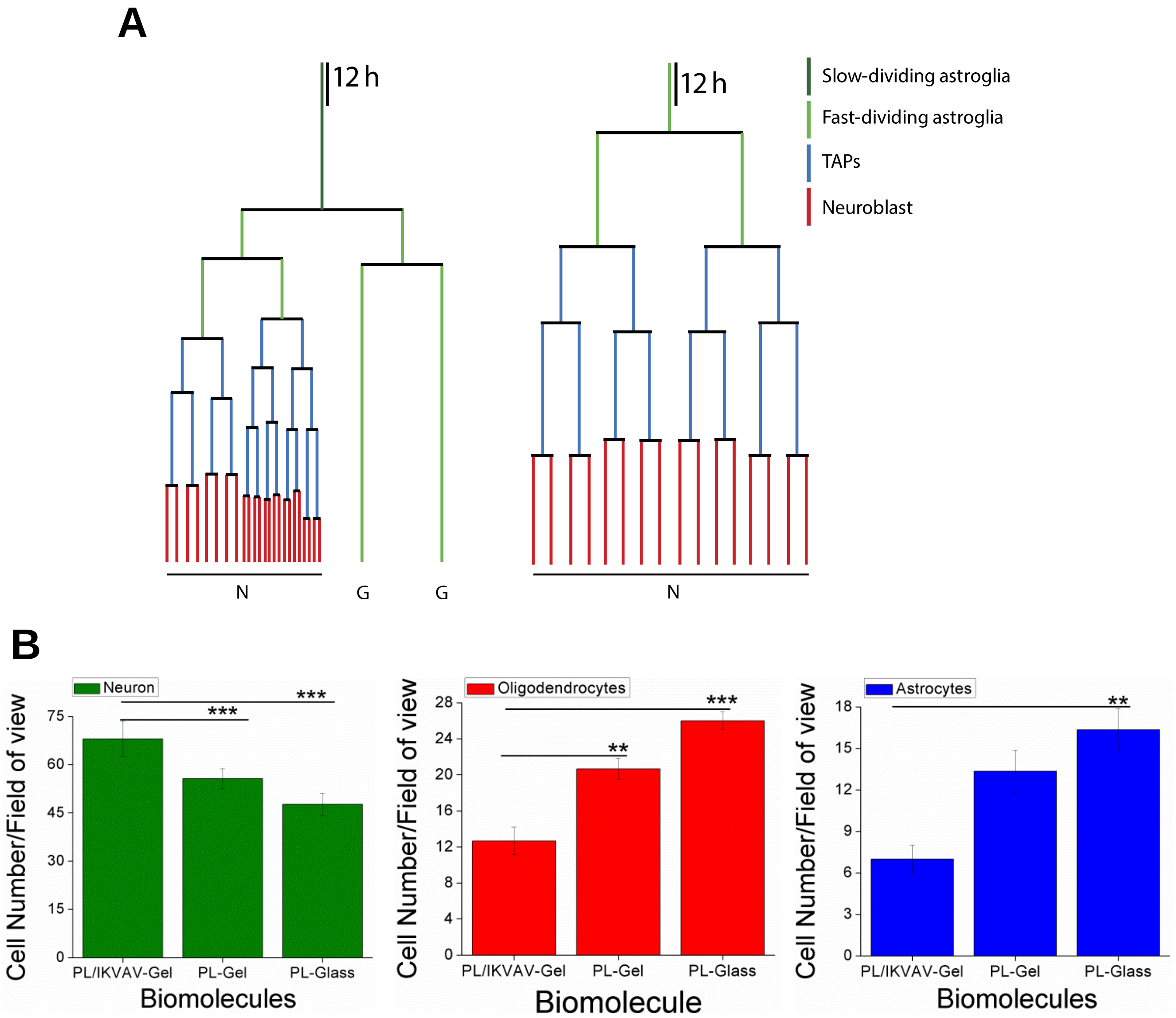

Figure SI3 related to figure 3. a) Examples of the standard lineage progression trees obtained by single cell tracking of aNSCs cultures isolated from the adult SEZ. Left: Asymmetric neurogenic tree which generates neuroblasts and quiescent GFAP positive cells in a potential aNSC self-renew event. Right: Symmetric neurogenic tree which generates only neuroblasts. Lineage trees depict the progression from slow-dividing astroglia (quiescent aNSCs) to activated fast-dividing aNSCs, transit amplifying progenitors and finally post-mitotic neuroblast. This representation allows to identify changes in rounds of division (X axis), cell cycle length (Y axis), cell fate decision or cell viability. According to the previous characterization, trees containing 5-6 rounds of division are typically asymmetric. In contrast, symmetric neurogenic trees are commonly restricted up to 4-5 rounds of amplifying divisions. **b)** Absolute numbers of neurons, oligodendrocytes and astrocytes from aNSCs cultures after 5 DIV on 20 kPa IKVAV/PL-gels, PL-gels and PL coated on glass (PL-glass). Data are represented as mean ± SD, (n = 3 independent experiments). Tukey post hoc test ** $p < 0.01$, *** $p < 0.001$.

Supplemental table

Quantity of Cross-linker (mg/mL)	Young's modulus E (kPa)
**0.04**	0.2 ± 0.1
**0.4**	2 ± 0.4
**17**	20 ± 0.3

Table S1 related to figure 1: Cross-linker ratios used to set PAAm-AA-MS gels with different stiffness. To obtain PA-gels of different stiffness, the amount of bisacrylamide used are indicated on the following table. Young's modulus is represented as mean ± SD

Experimental procedures

Cell-Substrate adhesion assay. The experiment was performed as previously described (Qin et al., 2005). Neurons were isolated from E14.5 cerebral cortex. Then cells were seeded on different substrate in the 24 wells plate and cultured in DMEM and 2% B27 medium for 48 hs. After 48 hs, cells were imaged and counted and then gentle washing for 3 times with HBSS to imaged and counted again (**Fig. SI2a**). The data were presented as the percentage of cells remaining in each washed well, compared with the unwashed control.

Primary antibodies used to differentiate cell types: Doublecortin (Abcam, ab18723-Cy3), SMI-312 for axonal neurofilaments (Abcam, ab24574-FITC), anti-beta III tubulin (SIGMA, T8660), GFAP (SIGMA G3893), MAP2 (Synaptic system, 188003) and Ng2 (Millipore, AB5320).

Patch clamp recordings: Cultures were placed in a recording chamber mounted on the stage of an upright microscope (Axio Imager.A2, Zeiss, Germany) equipped with epifluorescence and superfused (1 mL / min) with artificial cerebrospinal fluid solution (in mM): NaCl, 125; KCl, 2.5; NaHCO3, 25; CaCl2, 2; MgCl2., 1; NaH2PO4 1.25 and glucose 25, saturated with 5% CO2 and 95% O2, pH 7.4. Experiments were performed at 23-25 ºC. Cells were visualized with bright field and epifluorescence optics with a 40x (0.75 numerical aperture) objective. Images were taken and displayed using a software-operated CCD microscope camera (ORCA 0.3G, Hamamatsu). Patch-clamp whole-cell recordings were obtained with electrodes fabricated from thick borosilicate glasses pulled to a final resistance of 5–10 MΩ and filled with (in mM): K-gluconate, 125; NaCl, 5; Na2-ATP, 2; MgCl2, 2; EGTA, 10; HEPES, 10; biocytin, 10 and Alexa 488 hydrazide, 0.2 (Invitrogen); pH 7.4. Voltage-clamp recordings were performed using Axopatch 200B (Molecular Devices). Voltage steps were generated with Clampex10 (Molecular Devices) and data analysed with pClamp10 (Molecular Devices). Seal resistances were between 4 and 18 GΩ. In voltage clamp mode, voltage steps were applied from −70 mV holding potential to levels ranging from −100 to 40 mV in 10 mV steps. To subtract leak currents we used a P4 protocol provided by Clampex10 that allowed simultaneous storage of raw and leak subtracted data.

Biomolecule coupling on gel surface. Briefly, acrylamide (60 mg, 6%w/v), acrylic acid (6 µL, 0.6% w/v) and N,N-methylene-bis-acrylamide were dissolved in PBS (1 mL), and pH of solution was adjusted to pH 8. Methylsulfone comonomer (N-(4-(5-(methylsulfonyl)-1,3,4-oxadiazol-2-yl)phenyl)acrylamide) (4 mg, 0.4% w/v) was dissolved in DMF (100 µL) and mixed with above solution. The ammonium persulfate (initiator, 10% solution, 1/100 of total volume) and TEMED (catalyst, 1/1000 of total volume) was added to it under nitrogen. One drop of monomer solution (6 µL for 0.2, 10 µL for 2 and 20 kPa) was immediately placed on Sigmacote-coated glass slide and covered with 3-acryloxypropyl-trimethoxysilane functionalized coverslips. For binding PL, the gel was activated by 0.2 M EDC (N-(3-dimethylaminopropyl)-N′- ethylcarbodiimide hydrochloride), 0.1 M NHS (N-hydroxysuccinimide), 2-(N-morpho)-ethanesulfonic acid (0.1 M) and NaCl (0.5 M) for 15 min, followed by washing and 1 h coupling with (10 µgmL-1) PL solution in PBS. Bioconjugation of CSRARKQAASIKVAVSADR (IKVAV) (100 µgmL-1) was performed by placing a drop (20 µL) of peptide solution in PBS, on the gel surface and coupling for 1 h at r.t. For orthogonal coupling hydrogel was activated with EDC/NHS and incubated with PL solution (10 µgmL-1) for 1 h followed by washing and coupling with IKVAV solution (100 µgmL-1). The IKVAV/PL mixture was covalently bonded on APTES functionalized glass, activated by incubation with aq. glutaraldehyde solution (8%) for 6 h, subsequent washing and incubation with IKVAV/PL (100/10 µgmL-1) solution.

References

Qin, Y., Capaldo, C., Gumbiner, B.M., and Macara, I.G. (2005). The mammalian Scribble polarity protein regulates epithelial cell adhesion and migration through E-cadherin. The Journal of cell biology *171*, 1061-1071.
