# Supplementary material for: Bifunctional Hydrogels Containing the Laminin Motif IKVAV Promote Neurogenesis
Source: Stem Cell Reports. 2017 Oct 5;9(5):1432–40. doi: 10.1016/j.stemcr.2017.09.002 (PMC5829305; doi:10.1016/j.stemcr.2017.09.002)
Supplement: Document S2. Article plus Supplemental Information [file mmc2.pdf]

Stem Cell Reports
Report

OPEN ACCESS

# Bifunctional Hydrogels Containing the Laminin Motif IKVAV Promote Neurogenesis

Aleeza Farrukh,[1,8] Felipe Ortega,[2,3,4,8] Wenqiang Fan,[5,6,8] Nicolás Marichal,[5,6] Julieta I. Paez,[1] Benedikt Berninger,[5,6] Aránzazu del Campo,[1,7] and Marcelo J. Salierno[5,6,*]
[1]INM – Leibniz Institute for New Materials, Campus D2 2, 66123 Saarbrücken, Germany
[2]Biochemistry and Molecular Biology Department IV, Faculty of Veterinary Medicine, Complutense University, Madrid, Spain
[3]Institute of Neurochemistry (IUIN), 28040 Madrid, Spain
[4]Health Research Institute of the Hospital Clínico San Carlos (IdISSC), Madrid, Spain
[5]Institute of Physiological Chemistry, University Medical Center of the Johannes Gutenberg University Mainz, Hanns-Dieter-Hüsch-Weg 19, 55128 Mainz, Germany
[6]Focus Program Translational Neuroscience, Johannes Gutenberg University Mainz, 55131 Mainz, Germany
[7]Saarland University, Campus Saarbrücken D2 2, 66123 Saarbrücken, Germany
[8]Co-first author
*Correspondence: salierno@gmail.com
https://doi.org/10.1016/j.stemcr.2017.09.002

## SUMMARY

Engineering of biomaterials with specific biological properties has gained momentum as a means to control stem cell behavior. Here, we address the effect of bifunctionalized hydrogels comprising polylysine (PL) and a 19-mer peptide containing the laminin motif IKVAV (IKVAV) on embryonic and adult neuronal progenitor cells under different stiffness regimes. Neuronal differentiation of embryonic and adult neural progenitors was accelerated by adjusting the gel stiffness to 2 kPa and 20 kPa, respectively. While gels containing IKVAV or PL alone failed to support long-term cell adhesion, in bifunctional gels, IKVAV synergized with PL to promote differentiation and formation of focal adhesions containing $\beta_1$-integrin in embryonic cortical neurons. Furthermore, in adult neural stem cell culture, bifunctionalized gels promoted neurogenesis via the expansion of neurogenic clones. These data highlight the potential of synthetic matrices to steer stem and progenitor cell behavior via defined mechano-adhesive properties.

## INTRODUCTION

There is a surge of interest in designing hydrogels endowed with specific mechanical and chemical properties for steering cell behavior, including cell-fate decision and plasticity as well as organoid formation (Engler et al., 2006; Floren et al., 2016; Gjorevski et al., 2016; Her et al., 2013; Wen et al., 2014). Previously, we have studied lineage progression of neural stem cells (NSCs) from the adult subependymal zone (SEZ) using hard surfaces coated with polylysine (Costa et al., 2011; Ortega et al., 2011), leaving the question open whether the observed patterns of cell division and terminal differentiation within single clones can be altered by modifying the mechanical and chemical properties of the substrate. In fact, there is ample evidence that stem cell behavior is under strong influence of the molecular milieu provided by their niche (Theocharidis et al., 2014). One component shown to regulate the activated state of adult SEZ NSCs is the basement membrane glycoprotein laminin (Kokovay et al., 2010). Tashiro and colleagues identified a 19-mer peptide containing the pentapeptide IKVAV within the laminin $\alpha$1 chain as one of the principle sites in laminin to regulate cellular behavior (Sephel et al., 1989; Tashiro et al., 1989). IKVAV was found to enhance viability and maturation of neurons by binding to the $\beta_1$-integrin subunit (Agius et al., 1996; Li et al., 2014; Sur et al., 2012; Tashiro et al., 1989), rendering it a promising candidate for use in neuronal growth-stimulating materials.

Previously, we engineered bifunctionalized polyacrylamide gels (PA) allowing orthogonal coupling of thiol- and amine-containing ligands (Farrukh et al., 2016). Subsequently, we used this bifunctionalized PA to couple polylysine (via its amine group) and IKVAV (via its thiol group) independently to the substrate and studied its effect on neuronal survival and neurite outgrowth (Farrukh et al., 2017). In the present study, we aimed to optimize the mechanical properties of these bifunctionalized gels for culturing embryonic and adult neural progenitors by applying distinct stiffness regimes. Our work provides a rationale for further study on the consequences of culturing neural progenitors in microenvironments of enriched chemical functionality and mechanical properties.

## RESULTS

### Neurite Outgrowth of E14.5 Cortical Neurons Is Enhanced on 2 kPA IKVAV/PL Bifunctionalized Gels

We first evaluated the effect of bifunctionalized IKVAV/PL gels at different gel stiffness to determine the optimal mechanical conditions for neuronal differentiation of E14 embryonic cortical progenitors. Cells were seeded on substrates within the range of soft tissue stiffness (0.2, 2, and

 © 2017 The Authors.
This is an open access article under the CC BY license (http://creativecommons.org/licenses/by/4.0/).

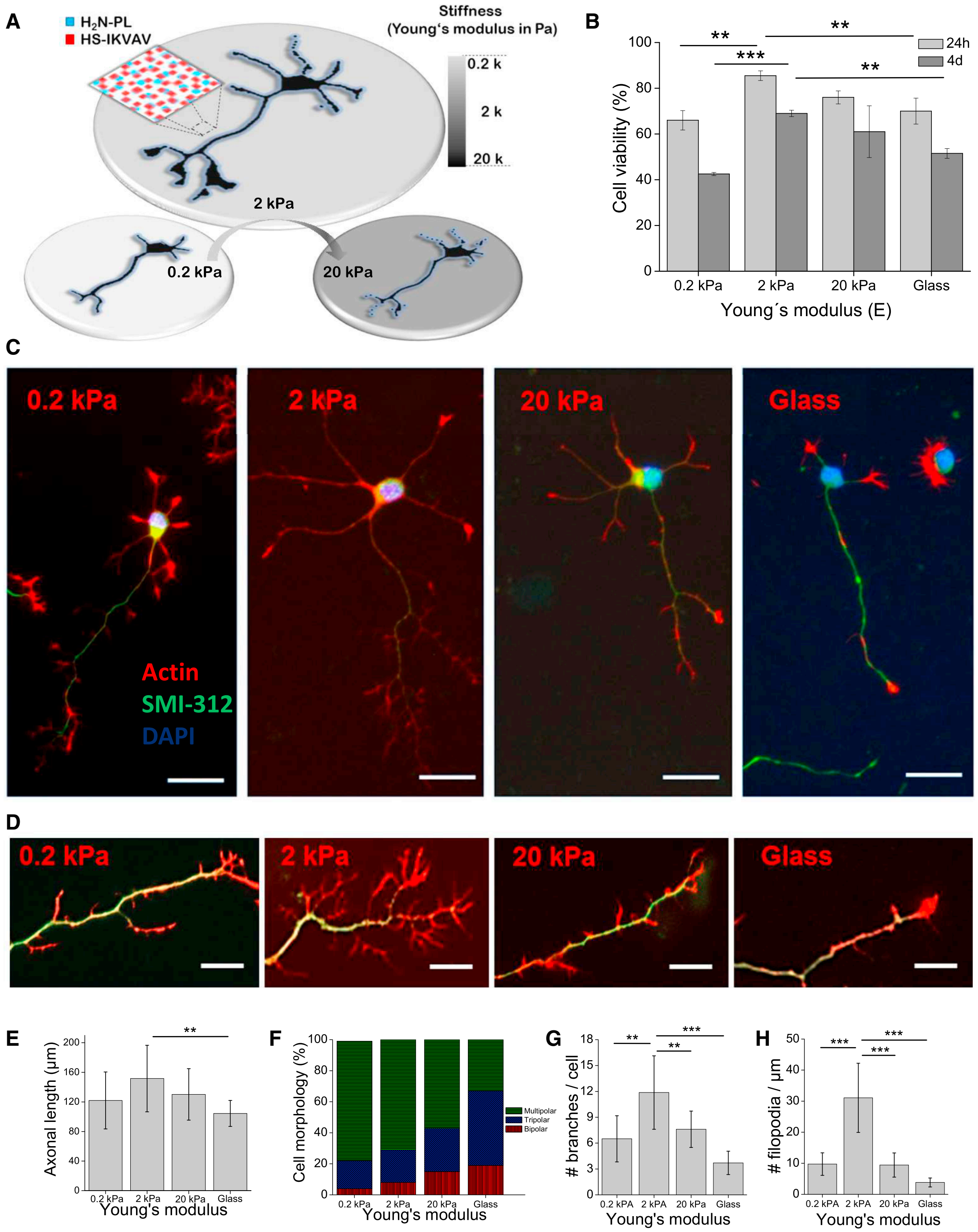


**Figure 1. Development of Cortical Progenitors on Bifunctionalized IKVAV/PL Substrates at Different Stiffnesses**

(A) Substrate platform based on PA gels containing two different biomolecules covalently immobilized with orthogonal coupling using thiols for IKVAV (red squares) and amines for PL (blue squares). In addition, hydrogels were adjusted at different Young's modulus E (kPa) in the range of soft tissue rigidities (i.e., 0.2, 2, and 20 kPa).

(B) Viability of cortical progenitor cells after 24 hr and 4 DIV cultured on bifunctionalized substrates at different gel stiffnesses. 2 kPa gels significantly improved neuronal viability (mean ± SD, ANOVA, Tukey-Kramer post hoc test $^{**}p < 0.01$, $^{***}p < 0.001$).

(C) Representative pictures of a single cortical progenitor after 4 DIV cultured on substrates at different stiffness (scale bar, 20 μm).

(D) The formation of actin filopodia (phalloidin reaction, in red) was appreciably incremented on 2 kPa IKVAV/PL gels, in particular along the axon (SMI-312 immunoreactivity, in green). Scale bar, 10 μm.

*(legend continued on next page)*

20 kPa) (Discher et al., 2005) as depicted in Figure 1A. Viability of cortical progenitors was significantly improved on 2 kPa IKVAV/PL gels compared with the other stiffness regimes (Figure 1B). Likewise, on day 4 of culture, neurite growth was enhanced on 2 kPa IKVAV/PL gels (Figure 1C), with an appreciable increment in actin filopodia (Figure 1D). Also, average axon length (identified by SMI-312 immunoreactivity, see Experimental Procedures) was significantly increased in 2 kPa IKVAV/PL gels compared with PL coating on glass (Figure 1E). Noticeably, the number of primary neurites displayed a reverse correlation with the degree of stiffness, with 0.2 kPA being most favorable for the protrusion of multiple primary processes (Figure 1F). However, the number of dendritic filopodia and secondary branches exhibited peak growth on 2 kPa gels compared with other levels of stiffness (Figures 1G and 1H). In fact, the number of dendritic filopodia was found to be the morphological feature that was most sensitive to mechanical changes. Further experiments demonstrated that neurite outgrowth was similar when the IKVAV containing 19mer was replaced by the complete laminin protein (Figures S1B–S1E). In contrast, substitution of IKVAV with another laminin motif cRGDfC (Yamada, 1991) on 2 kPa substrates showed no improvement of neurite outgrowth (Figures S1B–S1E), highlighting the specificity of the effect induced by the IKVAV motif.

### Bifunctionalized IKVAV/PL Gels Accelerate Maturation and Increase the Adhesiveness of Cortical Progenitors

We next addressed whether the enhanced morphological differentiation of early cortical neurons on 2 kPa IKVAV/PL gels correlated with increased physiological maturation. Using patch-clamp recording, we found that neurons on 2 kPA IKVAV/PL gels displayed well-developed fast voltage-gated inward currents (Figure 2A) typical of $Na^+$ channel activation ($I_{Na}$ maximum peak amplitude −278.4 ± 69 pA, n = 8 cells) similar to young neurons in acute or cultured embryonic cortical slices (Noctor et al., 2004). Remarkably, neuronal cultures grown on 2 kPa gels functionalized only with a single molecule, i.e., either IKVAV or PL alone (Figures 2B and 2C, respectively), displayed inward voltage-gated currents with noticeably lower amplitudes ($I_{Na}$ maximum peak amplitude −57.6 ± 34.3 pA, n = 5 cells, mean ± SEM, $p < 0.05$, Mann-Whitney U test). These data suggest a synergism of the IKVAV and PL functional groups to promote maturation of the intrinsic properties of cortical neurons.

### IKVAV Synergized with PL to Increase Adhesiveness and Focal Adhesions Containing β1-Integrin in Cortical Progenitors

Finally, we performed a cell-substrate adhesion assay (Humphries, 2001) to explore the adhesive properties of the bifunctionalized gels compared with single IKVAV or PL coupling. The cell ratio (i.e., cells after/cells before the washing procedure, see Figure S2A) declined significantly on all gel surfaces compared with PL-coated glass slides (Figure 2D). However, gels containing the IKVAV motif significantly increased cell adhesion compared with single PL coupling on gels, highlighting the adhesive effect of the short laminin sequence. In addition, as IKVAV is known to directly bind to the transmembrane receptor $\beta_1$-integrin (Agius et al., 1996; Nomizu et al., 1995), we quantified focal adhesions in cortical neurons 48 hr after plating. Immunocytochemistry revealed a significant increase in focal adhesions containing $\beta_1$-integrin on bifunctional IKVAV/PL substrates compared with monofunctional gels (Figure 2E). Intriguingly, $\beta_1$-integrin immunoreactivity appeared to be denser and expanded along the neurites extended on IKVAV/PL substrates (Figures 2F and S2B), consistent with the increased neurite outgrowth observed above.

### IKVAV/PL Bifunctionalized Gels Enhance Adult NSC Symmetric Neurogenic Divisions and Promote Overall Neurogenesis

Next, we investigated the effect of IKVAV/PL gels on adult neural stem cells (aNSCs) derived from the adult SEZ. In notable contrast to the results obtained with E14.5 cortical progenitors, aNSCs cultures showed reduced adherence and poorer survival on 0.2 and 2 kPa IKVAV/PL gels (data not shown). In further contrast, 20 kPa IKVAV/PL gels supported substantial cell survival of aNSC, allowing lineage progression toward neurogenesis. Therefore, we examined the generation of lineage trees (Figures 3A and S3A for a description of the lineage trees) via single-cell tracking of aNSCs on 20 kPa bifunctionalized IKVAV/PL gels and monofunctionalized 20 kPa PL gels (monofunctionalized 20 kPa IKVAV gels also failed to support substantial cell survival). Interestingly, aNSCs grown on bifunctional

(E) Average axon length was significantly increased in 2 kPa bifunctionalized gels compared with PL coated on glass.
(F) Distribution of number of primary neurites on cortical progenitor cells. The amount of neurites displayed a reverse correlation with the degree of gel stiffness.
(G and H) Average number of secondary branches (G) and dendritic filopodia (H) show a significantly increase on 2 kPa gel stiffness. Contrast were determined a *posteriori* against bifunctionalized 2 kPa gels which displayed the optimal mechanical conditions (n = 3 independent experiments, 20–25 fields of view).
Data in (E, G, and H) are represented as means ± SD, Tukey post hoc test (** $p < 0.01$, *** $p < 0.001$).

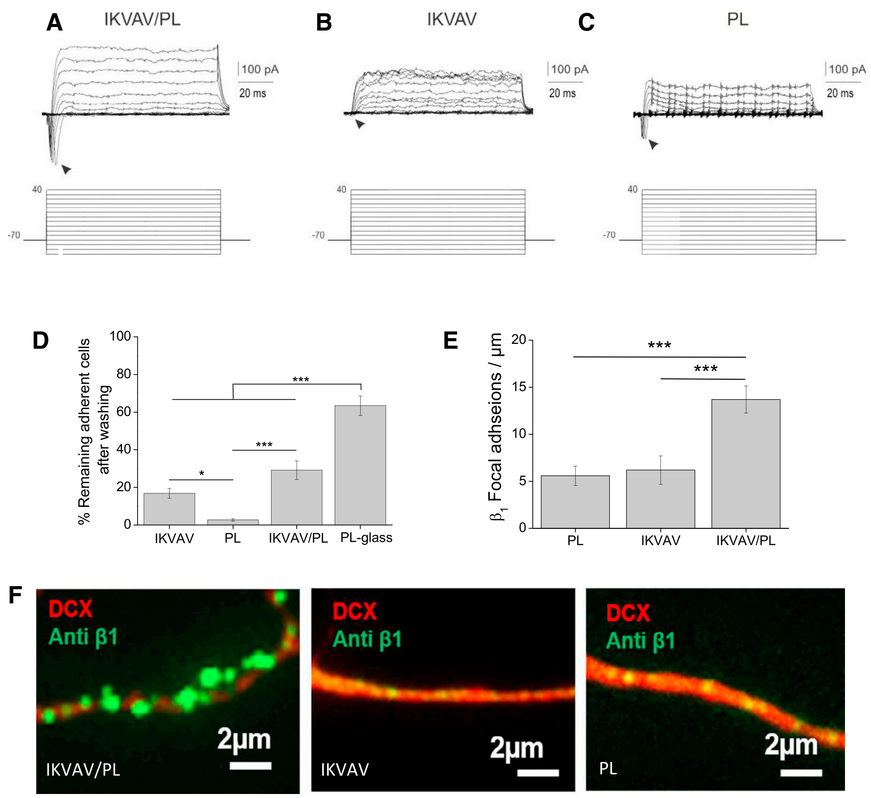


**Figure 2. 2 kPa IKVAV/PL Gels Accelerate Maturation and Enhance Adhesiveness of Cortical Neurons**

(A–C) Patch-clamp recording in a representative cell under different substrate conditions: IKVAV/PL (A), IKVAV (B), or PL (C). Graphics show leak-subtracted currents in response to a series of voltage steps. Arrowheads highlight voltage-dependent inward currents.

(D) Cell-substrate adhesion assay showed that cortical neurons significantly increase the adherence on IKVAV/PL gels after 48 hr. The average cell number for each condition before and after washing is shown in Figure S2A.

(E) Average focal adhesions containing $\beta_1$-integrin in neurons after 5 DIV. Substrates with IKVAV/PL bifunctionalization significantly increase $\beta_1$-integrin in comparison with single-gel coupling of IKVAV or PL.

(F) Focal adhesions containing $\beta_1$-integrin (green) along the neurites (DCX, red) substantially increase on IKVAV/PL bifunctionalized substrates (see also Figure S2B).

Data in (D) and (E) are represented as means ± SD, n = 3 independent experiments, 20–25 fields of view, Tukey post hoc test (*$p < 0.05$, ***$p < 0.001$).

IKVAV/PL gels displayed a higher percentage of symmetric lineage trees with more rounds of divisions (Figure 3B) leading to ~3 times increase in the number of neurons in comparison with PL gels (Figure 3C). Intriguingly, the effect of IKVAV ligand on promoting cell neurogenesis was further supported by a significant decrease of glial lineages in absolute numbers (i.e., astrocytes and oligodendrocytes) on IKVAV/PL gels in comparison with PL gels or PL coated on glass (Figures 3D and S3B).

### Maturation and Neurite Outgrowth of Adult NSCs Are Accelerated on 20 kPA IKVAV/PL Bifunctionalized Gels

Moreover, single-cell tracking showed that besides the increase in Map2-positive neurons on 20 kPa IKVAV/PL gels at the clonal level, these neurons also expressed axon-specific neurofilament identified by SMI-312 immunoreactivity (Figure 4A), while neurofilament expression was not detected at the same stage in neuronal clones grown on 20 kPA PL gels (Figure 4B). This suggests a higher degree of maturation of aNSC-derived neurons on bifunctionalized IKVAV/PL gels. In addition, neuronal survival was improved resulting in a higher percentage of viable progressing neurons (~50%) toward maturation (Figure 4C). In agreement with our data in cultures of cortical progenitors, aNSCs exhibited a significant increase in neurite outgrowth on IKVAV/PL gels compared with PL gels after 5 days in vitro (DIV) (e.g., neurite length, number, and branches) (Figures 4D–4F).

## DISCUSSION

Here, we demonstrate that PA gels containing IKVAV and PL are a valuable design to enhance neurogenesis and promote neurite outgrowth. Furthermore, we provide evidence for the importance of adjusting gel elasticity in a cell-type-specific manner. Notably, embryonic cortical progenitors and adult NSCs were optimally cultured on gels with different matrix elasticity (2 kPA and 20 kPA, respectively). Beyond the adjustment of the substrate stiffness, functionalization with the IKVAV motif appeared as an important pro-neurogenic factor, in agreement with recent studies which employed this motif in biomaterials to improve cultures of dorsal root ganglial cells, induced pluripotent stem cells-neural progenitor cells, and hNSCs (Berns et al., 2014; Lam et al., 2015; Li et al., 2014). Yet, bifunctionalization by orthogonally coupling with IKVAV and PL worked synergistically to boost neurogenic IKVAV

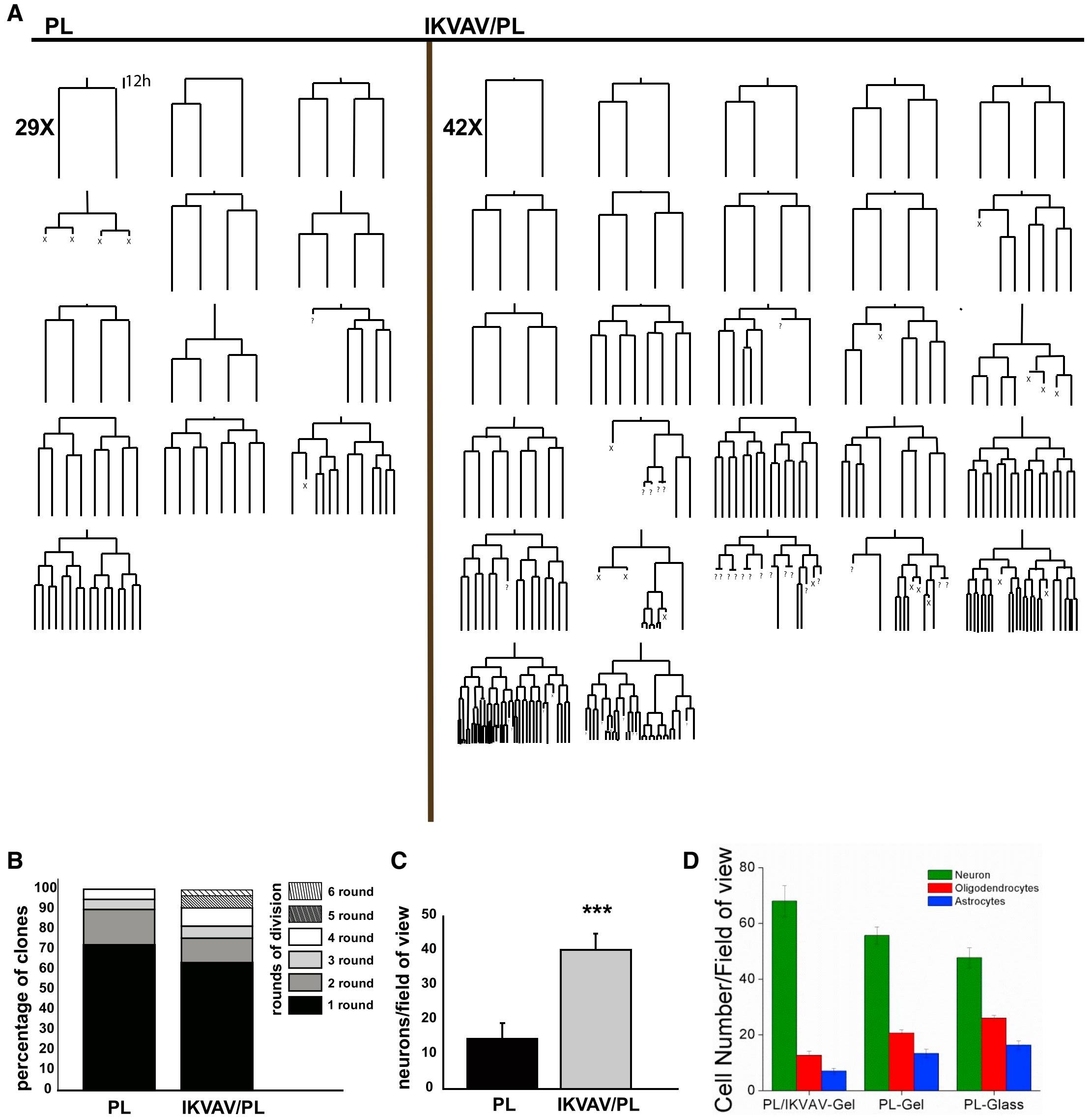


**Figure 3. Neurogenic Development of aNSCs Clones: Comparison of IKVAV/PL versus PL Gel Platforms at 20 kPa Stiffness**

(A) Lineage trees representing the clonal evolution of single aNSCs *in vitro*. The figure compares the trees tracked under PL gels (left) versus IKVAV/PL gels (right) at 20 kPa stiffness. Bifunctional gels produced a higher number of clones. Tree reconstructions showed mostly symmetric distribution with more rounds of divisions on IKVAV/PL gels.

(B) Histogram showing the fraction of clones per round of division. IKVAV/PL gels presented 6% of clones containing 5 rounds of division and 3% of the symmetric neurogenic clones with an unusual 6 rounds of division.

(C) Number of mature neurons per field of view after 5 DIV. t test (***$p < 0.001$).

(D) Average number for a particular cell type (neurons, astrocytes, and oligodendrocytes) per field of view on 20 kPa IKVAV/PL gels, 20 kPa PL gels, or PL coated on glass. Significant differences for each cell type between conditions are shown in Figure S3B. Data are represented as means ± SEM, n = 3 independent experiments.

properties while also promoting cell adhesion. IKVAV/PL synergism was found to increment focal adhesions containing $\beta_1$-integrin in embryonic cortical neurons, particularly on their neurites, providing a mechanistic explanation for the observed increase in overall cell adhesiveness and enhanced neurite outgrowth. This is in line with previous studies showing that $\beta_1$-integrin promotes neurogenesis and enhances proliferation upon expression of a constitutively active $\beta_1$-integrin in the embryonic chick mesencephalon (Long et al., 2016), and that ablation of β1-integrin leads to reduced neurogenesis from hippocampal NSCs (Brooker et al., 2016).

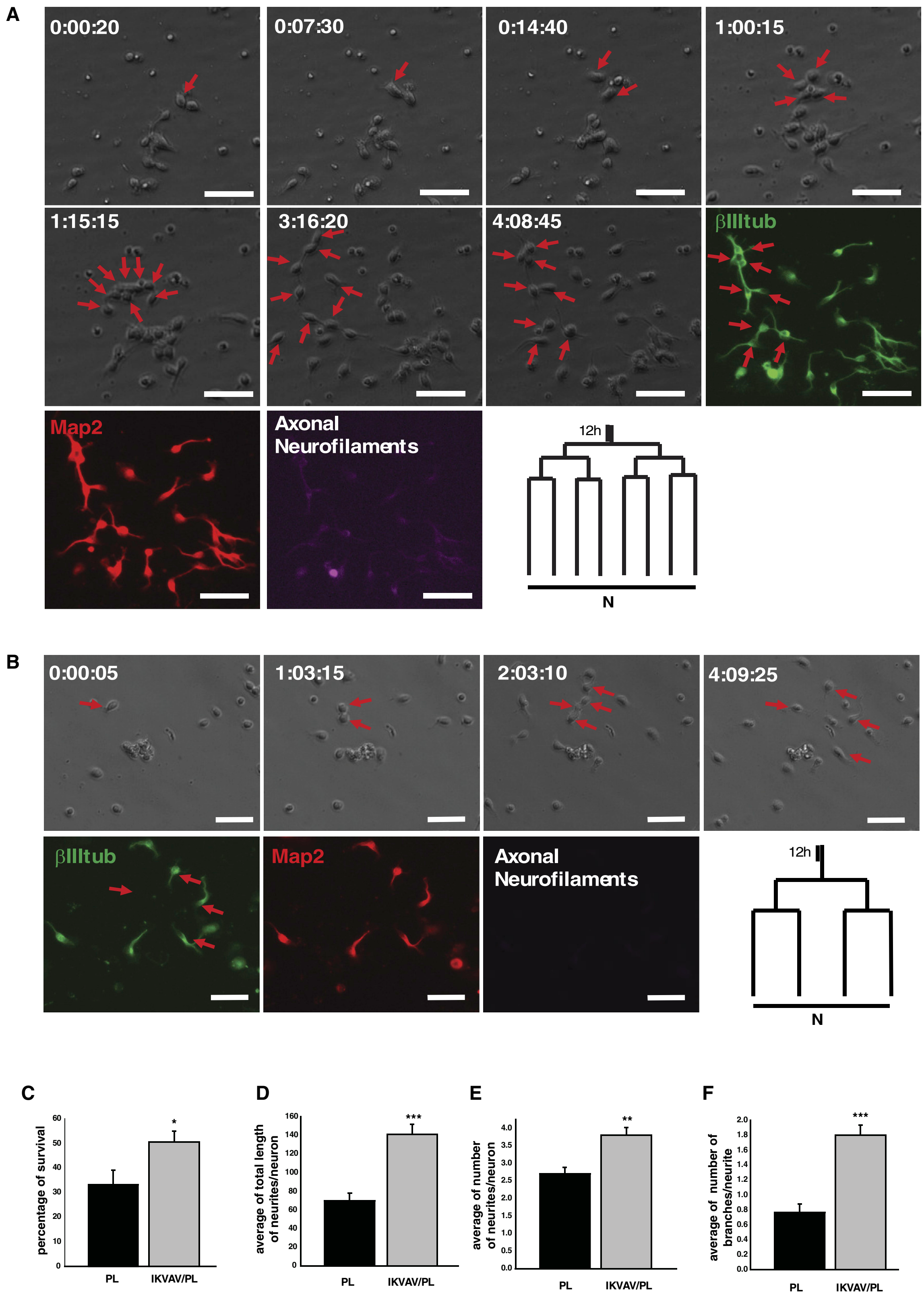


*(legend on next page)*

IKVAV/PL gels modulated lineage progression of the SEZ-derived aNSCs (Ortega et al., 2011; Ponti et al., 2013) by increasing the rounds of amplifying divisions. This is consistent with previous results showing that IKVAV increases proliferation of cultured human NSCs (Li et al., 2014). The IKVAV peptide is not only important for regulation of NSC proliferation but is also directly connected to the promotion of neuronal maturation and survival in hNSCs, murine cerebellar granule neurons, and Purkinje neurons (Li et al., 2014; Sur et al., 2012, 2014). In addition, our study showed reduced gliogenesis from adult NSCs cultured on IKVAV/PL gels. This is in line with a recent study demonstrating that the injection of the IKVAV epitope into the ependymal region induces high levels of $\beta_1$-integrin in ependymal stem cells *in vivo*, resulting in a reduction of astrogliogenesis (Pan et al., 2014). Previously, pioneering work showed that self-assembly of nanotubes from amphiphilic peptides containing IKVAV inhibited glial differentiation in NSC cultures (Silva et al., 2004); yet, this differentiation suppressive effect was not observed when applying the IKVAV peptide in solution in the absence of the charges provided by the remainder of the amphiphilic peptide. In a similar manner, only bifunctionalization of IKVAV with PL in PA gels succeeded in significantly improving neuronal maturation and survival in comparison with monofunctionalized gels, stressing that IKVAV mimetic peptide should be inserted in a favorable environment in order to boost neurogenesis.

In summary, the use of bifunctionalized IKVAV/PL gels is a novel strategy to enhance neurogenesis *in vitro*. Moreover, optimization of the substrate elasticity enhanced the biological action of IKVAV. Thus, PA gels represent platforms with highly tunable mechanical properties (Kandow et al., 2007) that cover the entire range of elastic modulus encountered in animal soft tissues (Yeung et al., 2005). The platform presented here allows modulation of mechanical properties while defining adhesive properties via orthogonal coupling of IKVAV and PL to govern cellular behavior. Future developments of this strategy may allow improved control of organoid development as has been recently proved in the case of intestinal stem cells (Gjorevski et al., 2016). Here, we demonstrate that cell-surface interactions together with appropriate mechanical properties can increase cell survival, direct cell fate, and boost differentiation, highlighting the usefulness of engineered substrates for controlling stem cell cultures.

## EXPERIMENTAL PROCEDURES

### Embryonic and Adult Cortical Progenitor Isolation and Culture

Cerebral cortex from E14.5 of C57BL/6 mice was digested in 0.5% trypsin EDTA (GIBCO) for 15 min at 37°C. Trypsin was inactivated with DMEM (GIBCO) and 10% fetal bovine serum (Hyclone) and gently triturated with a 5 mL disposable pipette to get a single cell. After centrifuging at 1,000 rpm for 5 min, cell pellets were re-suspended with 1 mL of differentiating medium (DMEM +2% B27 supplement from Invitrogen). Substrates were placed in a 24-well plate seeding $5 \times 10^4$ cells in each well. For adult NSC culture, SEZ cultures were prepared from the lateral wall of the adult SEZ of young adult (8–12 weeks) C57/BL6 mice as previously described (Ortega et al., 2011).

### Live-Cell Imaging

A cell observer (Zeiss) and a Nikon TE-2000 were used for time lapse studies at a constant temperature of 37°C and 8% $CO_2$. Images were acquired every 5 min using a 20× PH objective (Zeiss or Nikon), an AxioCamHRm/ZYLA from ANDOR cameras, and Zeiss AxioVision 4.7/NIS-elements from Nikon software.

### Single-Cell Clonal Trees

Trees were reconstructed from single-cell tracking using a self-written computer program (tTt) (Hilsenbeck et al., 2016). The identity of the progeny was determined by post-imaging ICC as previously described (Ortega et al., 2011).

### Cell Viability Analysis

Cell viability (%) = cells at $t_{(24\ \mathrm{hr\ or\ 4\ days})}$/cells at $t_0$. Cell density was obtained from time-lapse videos starting 2 hr after seeding ($t_0$).

### Immunofluorescence and Focal Adhesion Quantification

Cells were fixed with prewarmed 4% (v/v) paraformaldehyde for 15 min, washed with PBS, permeabilized using 0.5% (v/v) Triton X-100 in PBS for 15 min and washed with PBS. Cells were stained with the appropriate primary antibodies (see Supplemental Information) prepared in 20% (v/v) horse serum (Gibco) for either 2 hr at room temperature or overnight at +4°C, washed with PBS and incubated with secondary antibodies prepared in 30% horse serum and PBS for at least 60 min. The nucleus was stained using

**Figure 4. IKVAV/PL Gels Increase aNSCs Survival, Maturation, and Neurite Outgrowth**
(A and B) Pictures showing the time lapse of a representative aNSC clone (day:hour:minute) on 20 kPa IKVAV/PL gels (A) or PL gels (B). Red arrows indicate the clonal expansion that was represented in a lineage tree for each case. The last three pictures show the post-imaging staining for neuronal markers βIII tub (green), Map2 (red), and the axonal neurofilament marker SMI-312 (purple).
(C–F) Percentage of viable cells after 5 DIV. Analysis of neurite outgrowth: average length of neurites per neuron (D), average number of neurites per neuron (E), and average number of secondary branches per neurite (F). Data are represented as means ± SEM, t test (*$p < 0.05$, **$p < 0.01$, ***$p < 0.001$), n = 3 independent experiments.
Scale bar, 50 μm.

DAPI. Individual focal adhesions were identified using anti-$\beta_1$-integrin antibody (Abcam, ab95623) and counted using the analyze particles built-in function of ImageJ.

### Electrophysiological Recordings

Patch-clamp whole-cell recordings were made using an Axopatch 200B (Molecular Devices) amplifier, which allowed voltage-clamp measurements (see Patch-clamp recording in Supplemental Information for more detail).

### Hydrogel Preparation

Gel stiffness was prepared by following a previously reported protocol (Farrukh et al., 2016, 2017) by free-radical copolymerization of methylsulfone acrylate, acrylamide, and acrylic acid with varying amount of N,N′-methylene-bis-acrylamide crosslinker to set PA gels at 0.2, 2, and 20 kPa (see Table S1). The physical properties of the gels obtained coincide with the literature (Farrukh et al., 2016, 2017; Zouani et al., 2013). Both binding strategies are stable under physiological conditions and also in the presence of cell culture medium, imparting uniform surface distribution of ligand.

### Statistical Analysis

All experiments comprised at least three independent experimental batches. Data are expressed as means ± SD. Statistical analysis for parametric results used the t test, ANOVA with a post hoc Tukey or Mann-Whitney U test. Statistical significance was determined between groups from $p < 0.05$.

## SUPPLEMENTAL INFORMATION

Supplemental Information includes Supplemental Experimental Procedures, three figures, and one table and can be found with this article online at https://doi.org/10.1016/j.stemcr.2017.09.002.

## AUTHOR CONTRIBUTIONS

A.F. prepared all substrates, designed and conducted experiments, and analyzed data. F.O. designed and conducted experiments and analyzed the aNSC data. W.F. conducted experiments, analyzed data, and performed cortical progenitor cultures. N.M. conducted the electrophysiological experiments. J.I.P., A.C., and B.B. provided critical revisions to the manuscript. M.J.S. designed experiments, analyzed data, and wrote the manuscript. All authors approved the final manuscript.


## ACKNOWLEDGMENTS

A.F. received financial support from the Max-Planck Graduate Center during her PhD program. F.O. was supported by research grants Red de excelencia Consolider-Ingenio Spanish Ion Channel Initiative (BFU2015-70067REDC), MEC (BFU2014-53654-P), BRADECM (S2013/ICE-2958), UCM-Santander (PR26/16-18B-3), Fundación Ramon Areces (PR2018/16-02), and the Ramon y Cajal Program of the Spanish Ministry of Economy and Competitiveness (MEC) (RYC-2013-13290). W.F. was supported by a fellowship of the China Scholarship Council and N.M. by a Human Frontier Science Program Long-Term Fellowship. M.S. acknowledges internal funding ("Stufe 1") from Johannes Gutenberg University Mainz. This work was supported by a NEURON ERA-NET grant (01EW1604) to B.B.

Received: December 8, 2016
Revised: September 1, 2017
Accepted: September 1, 2017
Published: October 5, 2017


## REFERENCES


Agius, E., Sagot, Y., Duprat, A.M., and Cochard, P. (1996). Antibodies directed against the beta 1-integrin subunit and peptides containing the IKVAV sequence of laminin perturb neurite outgrowth of peripheral neurons on immature spinal cord substrata. Neuroscience *71*, 773–786.

Berns, E.J., Sur, S., Pan, L., Goldberger, J.E., Suresh, S., Zhang, S., Kessler, J.A., and Stupp, S.I. (2014). Aligned neurite outgrowth and directed cell migration in self-assembled monodomain gels. Biomaterials *35*, 185–195.

Brooker, S.M., Bond, A.M., Peng, C.Y., and Kessler, J.A. (2016). beta1-integrin restricts astrocytic differentiation of adult hippocampal neural stem cells. Glia *64*, 1235–1251.

Costa, M.R., Ortega, F., Brill, M.S., Beckervordersandforth, R., Petrone, C., Schroeder, T., Gotz, M., and Berninger, B. (2011). Continuous live imaging of adult neural stem cell division and lineage progression in vitro. Development *138*, 1057–1068.

Discher, D.E., Janmey, P., and Wang, Y.L. (2005). Tissue cells feel and respond to the stiffness of their substrate. Science *310*, 1139–1143.

Engler, A.J., Sen, S., Sweeney, H.L., and Discher, D.E. (2006). Matrix elasticity directs stem cell lineage specification. Cell *126*, 677–689.

Farrukh, A., Paez, J.I., Salierno, M., and del Campo, A. (2016). Bioconjugating thiols to poly(acrylamide) gels for cell culture using methylsulfonyl co-monomers. Angew. Chem. *55*, 2092–2096.

Farrukh, A., Paez, J.I., Salierno, M., Fan, W., Berninger, B., and Del Campo, A. (2017). Bifunctional poly(acrylamide) hydrogels through orthogonal coupling chemistries. Biomacromolecules *18*, 906–913.

Floren, M., Bonani, W., Dharmarajan, A., Motta, A., Migliaresi, C., and Tan, W. (2016). Human mesenchymal stem cells cultured on silk hydrogels with variable stiffness and growth factor differentiate into mature smooth muscle cell phenotype. Acta Biomater. *31*, 156–166.

Gjorevski, N., Sachs, N., Manfrin, A., Giger, S., Bragina, M.E., Ordonez-Moran, P., Clevers, H., and Lutolf, M.P. (2016). Designer matrices for intestinal stem cell and organoid culture. Nature *539*, 560–564.

Her, G.J., Wu, H.C., Chen, M.H., Chen, M.Y., Chang, S.C., and Wang, T.W. (2013). Control of three-dimensional substrate stiffness to manipulate mesenchymal stem cell fate toward neuronal or glial lineages. Acta Biomater. *9*, 5170–5180.

Hilsenbeck, O., Schwarzfischer, M., Skylaki, S., Schauberger, B., Hoppe, P.S., Loeffler, D., Kokkaliaris, K.D., Hastreiter, S., Skylaki, E., Filipczyk, A., et al. (2016). Software tools for single-cell tracking

and quantification of cellular and molecular properties. Nat. Biotechnol. *34*, 703–706.

Humphries, M.J. (2001). Cell-substrate adhesion assays. Curr. Protoc. Cell Biol. *Chapter 9*, Unit 9.1.

Kandow, C.E., Georges, P.C., Janmey, P.A., and Beningo, K.A. (2007). Polyacrylamide hydrogels for cell mechanics: steps toward optimization and alternative uses. Methods Cell Biol. *83*, 29–46.

Kokovay, E., Goderie, S., Wang, Y., Lotz, S., Lin, G., Sun, Y., Roysam, B., Shen, Q., and Temple, S. (2010). Adult SVZ lineage cells home to and leave the vascular niche via differential responses to SDF1/CXCR4 signaling. Cell Stem Cell *7*, 163–173.

Lam, J., Carmichael, S.T., Lowry, W.E., and Segura, T. (2015). Hydrogel design of experiments methodology to optimize hydrogel for iPSC-NPC culture. Adv. Healthc. Mater. *4*, 534–539.

Li, X., Liu, X., Josey, B., Chou, C.J., Tan, Y., Zhang, N., and Wen, X. (2014). Short laminin peptide for improved neural stem cell growth. Stem Cells Transl. Med. *3*, 662–670.

Long, K., Moss, L., Laursen, L., Boulter, L., and ffrench-Constant, C. (2016). Integrin signalling regulates the expansion of neuroepithelial progenitors and neurogenesis via Wnt7a and Decorin. Nat. Commun. *7*, 10354.

Noctor, S.C., Martinez-Cerdeno, V., Ivic, L., and Kriegstein, A.R. (2004). Cortical neurons arise in symmetric and asymmetric division zones and migrate through specific phases. Nat. Neurosci. *7*, 136–144.

Nomizu, M., Kim, W.H., Yamamura, K., Utani, A., Song, S.Y., Otaka, A., Roller, P.P., Kleinman, H.K., and Yamada, Y. (1995). Identification of cell binding sites in the laminin alpha 1 chain carboxyl-terminal globular domain by systematic screening of synthetic peptides. J. Biol. Chem. *270*, 20583–20590.

Ortega, F., Costa, M.R., Simon-Ebert, T., Schroeder, T., Gotz, M., and Berninger, B. (2011). Using an adherent cell culture of the mouse subependymal zone to study the behavior of adult neural stem cells on a single-cell level. Nat. Protoc. *6*, 1847–1859.

Pan, L., North, H.A., Sahni, V., Jeong, S.J., McGuire, T.L., Berns, E.J., Stupp, S.I., and Kessler, J.A. (2014). beta1-Integrin and integrin linked kinase regulate astrocytic differentiation of neural stem cells. PLoS One *9*, e104335.

Ponti, G., Obernier, K., Guinto, C., Jose, L., Bonfanti, L., and Alvarez-Buylla, A. (2013). Cell cycle and lineage progression of neural progenitors in the ventricular-subventricular zones of adult mice. Proc. Natl. Acad. Sci. USA *110*, E1045–E1054.

Sephel, G.C., Tashiro, K.I., Sasaki, M., Greatorex, D., Martin, G.R., Yamada, Y., and Kleinman, H.K. (1989). Laminin A chain synthetic peptide which supports neurite outgrowth. Biochem. Biophys. Res. Commun. *162*, 821–829.

Silva, G.A., Czeisler, C., Niece, K.L., Beniash, E., Harrington, D.A., Kessler, J.A., and Stupp, S.I. (2004). Selective differentiation of neural progenitor cells by high-epitope density nanofibers. Science *303*, 1352–1355.

Sur, S., Guler, M.O., Webber, M.J., Pashuck, E.T., Ito, M., Stupp, S.I., and Launey, T. (2014). Synergistic regulation of cerebellar Purkinje neuron development by laminin epitopes and collagen on an artificial hybrid matrix construct. Biomater. Sci. *2*, 903–914.

Sur, S., Pashuck, E.T., Guler, M.O., Ito, M., Stupp, S.I., and Launey, T. (2012). A hybrid nanofiber matrix to control the survival and maturation of brain neurons. Biomaterials *33*, 545–555.

Tashiro, K., Sephel, G.C., Weeks, B., Sasaki, M., Martin, G.R., Kleinman, H.K., and Yamada, Y. (1989). A synthetic peptide containing the IKVAV sequence from the A chain of laminin mediates cell attachment, migration, and neurite outgrowth. J. Biol. Chem. *264*, 16174–16182.

Theocharidis, U., Long, K., ffrench-Constant, C., and Faissner, A. (2014). Regulation of the neural stem cell compartment by extracellular matrix constituents. Prog. Brain Res. *214*, 3–28.

Wen, J.H., Vincent, L.G., Fuhrmann, A., Choi, Y.S., Hribar, K.C., Taylor-Weiner, H., Chen, S., and Engler, A.J. (2014). Interplay of matrix stiffness and protein tethering in stem cell differentiation. Nat. Mater. *13*, 979–987.

Yamada, K.M. (1991). Adhesive recognition sequences. J. Biol. Chem. *266*, 12809–12812.

Yeung, T., Georges, P.C., Flanagan, L.A., Marg, B., Ortiz, M., Funaki, M., Zahir, N., Ming, W., Weaver, V., and Janmey, P.A. (2005). Effects of substrate stiffness on cell morphology, cytoskeletal structure, and adhesion. Cell Motil. Cytoskeleton *60*, 24–34.

Zouani, O.F., Kalisky, J., Ibarboure, E., and Durrieu, M.C. (2013). Effect of BMP-2 from matrices of different stiffnesses for the modulation of stem cell fate. Biomaterials *34*, 2157–2166.

Stem Cell Reports, Volume *9*

## Supplemental Information

# Bifunctional Hydrogels Containing the Laminin Motif IKVAV Promote Neurogenesis

**Aleeza Farrukh, Felipe Ortega, Wenqiang Fan, Nicolás Marichal, Julieta I. Paez, Benedikt Berninger, Aránzazu del Campo, and Marcelo J. Salierno**

Supplemental figures

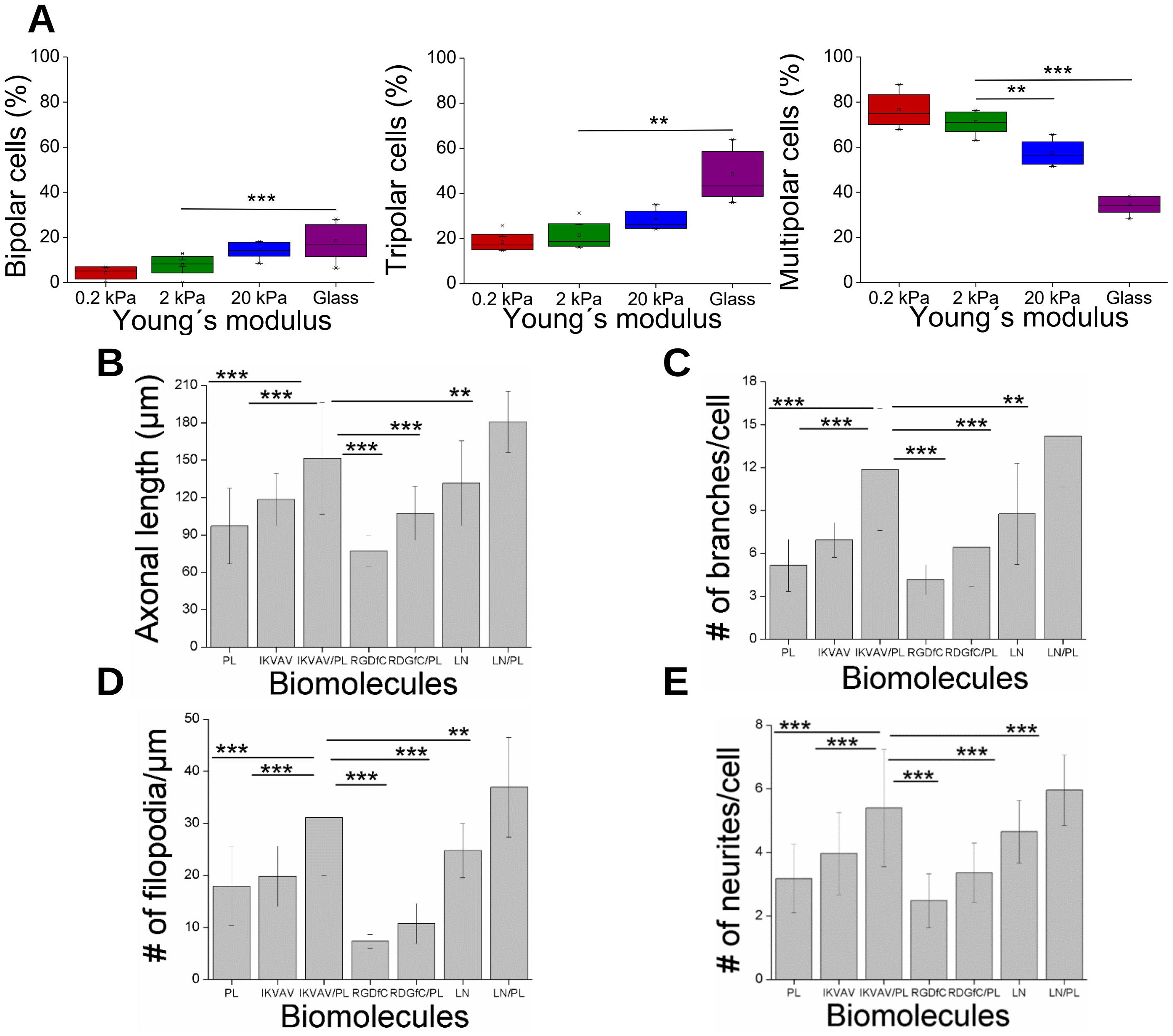


**Figure SI1 related to figure 1. a)** Quantification of average cell main processes depicting morphological changes in cortical progenitor population on IKVAV/PL-gels at different stiffness after 5 DIV: 2 neurites (bipolar), 3 neurites (tripolar) and >4 neurites (multipolar) (n = 54-60 cells, IQR: 25-75 percentile; whiskers: 1.5 x IQR, Kruskal-Wallis, Dunn's test ** $p < 0.01$, *** $p < 0.001$). Rounded cells were not included and there were no significant number of unipolar cells to be considered. Comparative analysis of matrix derived peptide sequences on eCPCs. 2 kPa-gels were functionalized with laminin and laminin-derived recognition sequences (cRGDfC, IKVAV) with or without PL. After 5 DIV morphological changes were compared: axonal length **(b)**, number of branches **(c)**, number of filopodia **(d)** and number of neurites **(e)**. For b to e data are represented as mean ± SD (n = 3 independent experiments), ANOVA, Tukey-Kramer post hoc test ** $p < 0.01$, *** $p < 0.001$.

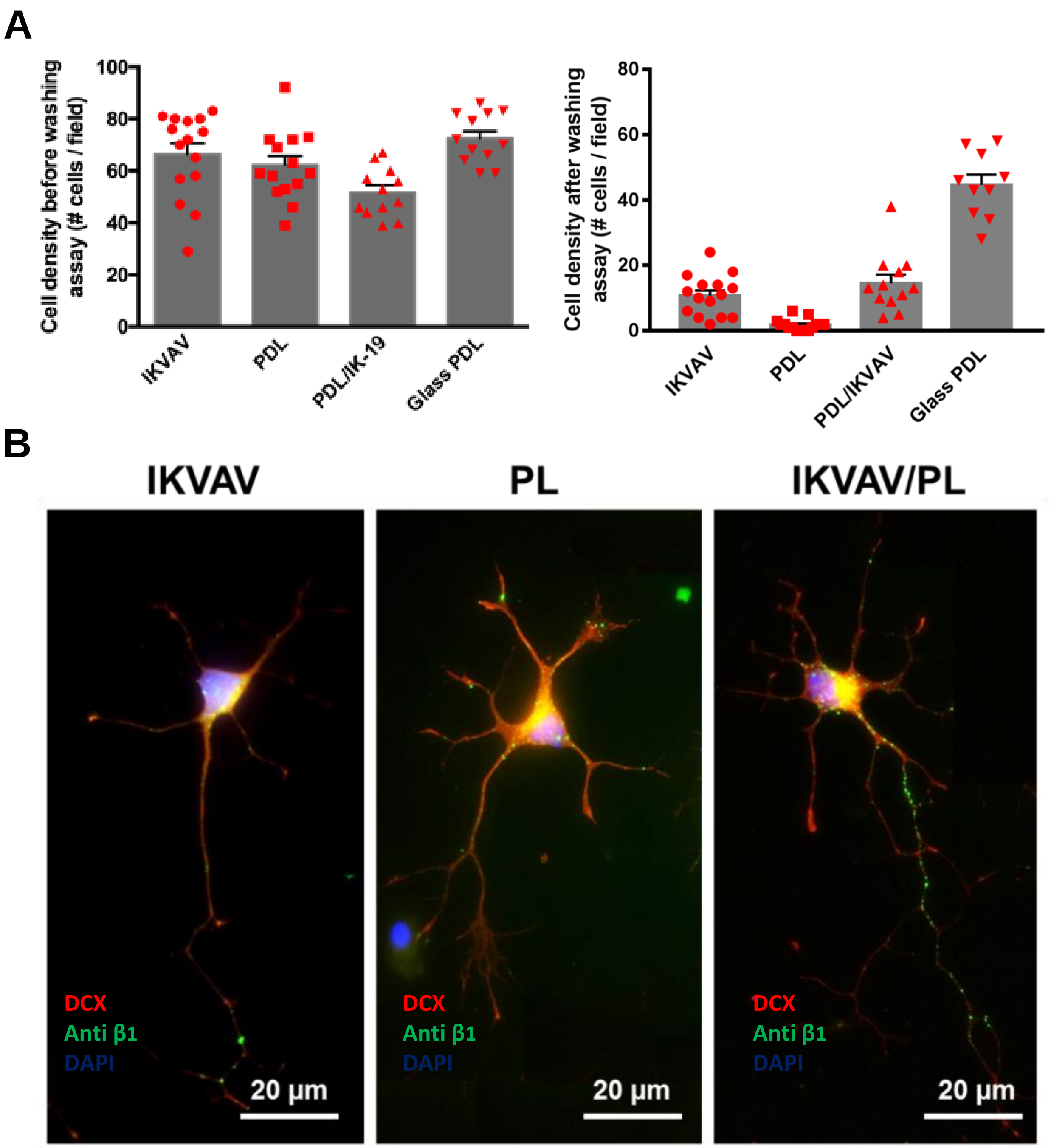


**Figure SI2 related to figure 2. a)** Average cell density before and after performing cell-substrate adhesion assay on each conditions. There were no significant differences between treatments before the test. Data are represented as mean ± SD (n = 3 independent experiments). **b)** Representative images of neurons after 5 DIV, stained with anti-β1 (green), DAPI (blue) and DCX (red) showing an increased density of focal adhesion containing β1 integrins in cells on IKVAV/PL-gels.

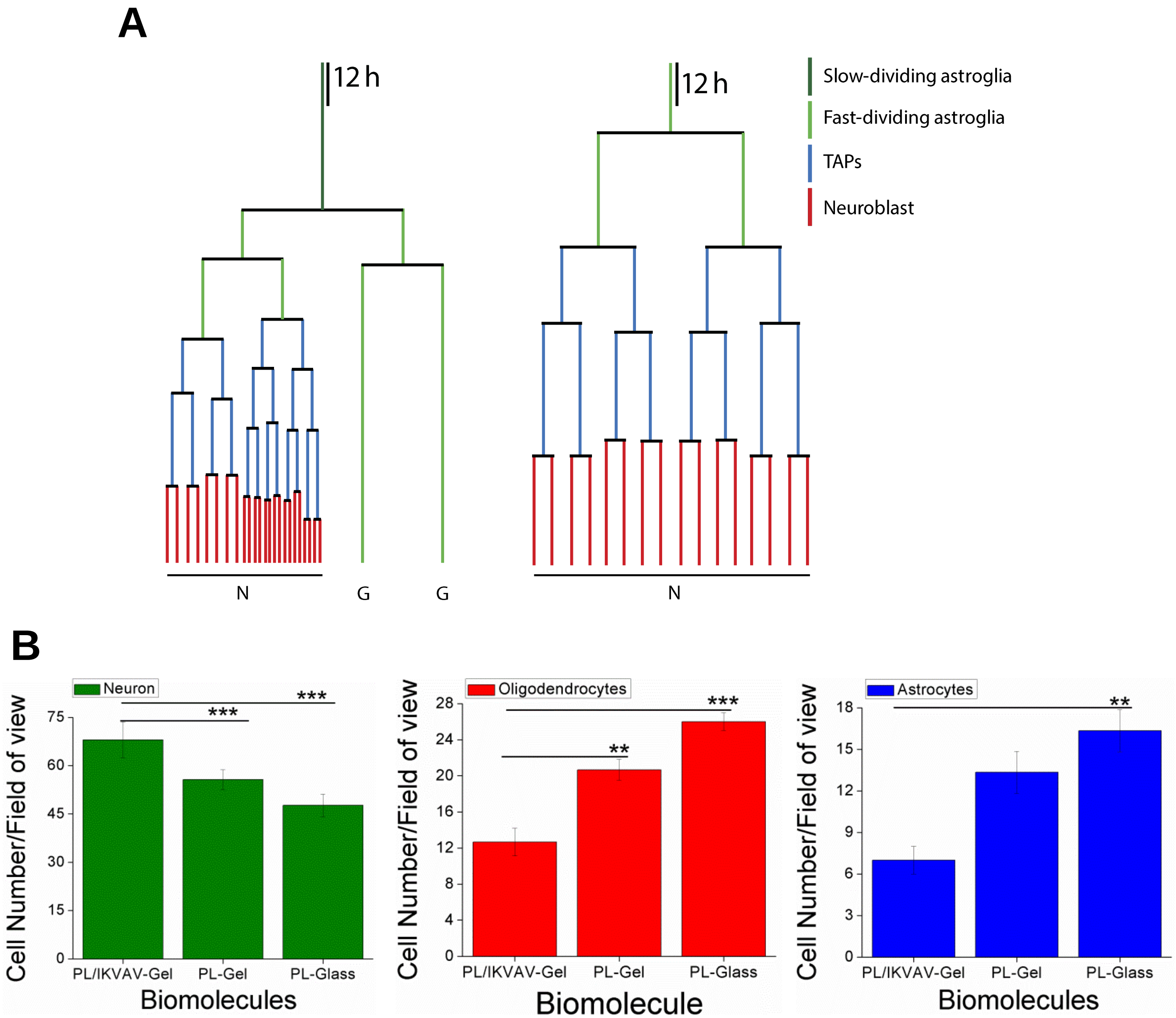


**Figure SI3 related to figure 3. a)** Examples of the standard lineage progression trees obtained by single cell tracking of aNSCs cultures isolated from the adult SEZ. Left: Asymmetric neurogenic tree which generates neuroblasts and quiescent GFAP positive cells in a potential aNSC self-renew event. Right: Symmetric neurogenic tree which generates only neuroblasts. Lineage trees depict the progression from slow-dividing astroglia (quiescent aNSCs) to activated fast-dividing aNSCs, transit amplifying progenitors and finally post-mitotic neuroblast. This representation allows to identify changes in rounds of division (X axis), cell cycle length (Y axis), cell fate decision or cell viability. According to the previous characterization, trees containing 5-6 rounds of division are typically asymmetric. In contrast, symmetric neurogenic trees are commonly restricted up to 4-5 rounds of amplifying divisions. **b)** Absolute numbers of neurons, oligodendrocytes and astrocytes from aNSCs cultures after 5 DIV on 20 kPa IKVAV/PL-gels, PL-gels and PL coated on glass (PL-glass). Data are represented as mean ± SD, (n = 3 independent experiments). Tukey post hoc test ** $p < 0.01$, *** $p < 0.001$.

Supplemental table

| Quantity of Cross-linker (mg/mL) | Young's modulus E (kPa) |
| --- | --- |
| **0.04** | 0.2 ± 0.1 |
| **0.4** | 2 ± 0.4 |
| **17** | 20 ± 0.3 |

**Table S1 related to figure 1: Cross-linker ratios used to set PAAm-AA-MS gels with different stiffness.** To obtain PA-gels of different stiffness, the amount of bisacrylamide used are indicated on the following table. Young's modulus is represented as mean ± SD

# Experimental procedures

**Cell-Substrate adhesion assay.** The experiment was performed as previously described (Qin et al., 2005). Neurons were isolated from E14.5 cerebral cortex. Then cells were seeded on different substrate in the 24 wells plate and cultured in DMEM and 2% B27 medium for 48 hs. After 48 hs, cells were imaged and counted and then gentle washing for 3 times with HBSS to imaged and counted again (**Fig. SI2a**). The data were presented as the percentage of cells remaining in each washed well, compared with the unwashed control.

**Primary antibodies used to differentiate cell types**: Doublecortin (Abcam, ab18723-Cy3), SMI-312 for axonal neurofilaments (Abcam, ab24574-FITC), anti-beta III tubulin (SIGMA, T8660), GFAP (SIGMA G3893), MAP2 (Synaptic system, 188003) and Ng2 (Millipore, AB5320).

**Patch clamp recordings:** Cultures were placed in a recording chamber mounted on the stage of an upright microscope (Axio Imager.A2, Zeiss, Germany) equipped with epifluorescence and superfused (1 mL / min) with artificial cerebrospinal fluid solution (in mM): NaCl, 125; KCl, 2.5; NaHCO3, 25; CaCl2, 2; MgCl2., 1; NaH2PO4 1.25 and glucose 25, saturated with 5% CO2 and 95% O2, pH 7.4. Experiments were performed at 23-25 ºC. Cells were visualized with bright field and epifluorescence optics with a 40x (0.75 numerical aperture) objective. Images were taken and displayed using a software-operated CCD microscope camera (ORCA 0.3G, Hamamatsu). Patch-clamp whole-cell recordings were obtained with electrodes fabricated from thick borosilicate glasses pulled to a final resistance of 5–10 MΩ and filled with (in mM): K-gluconate, 125; NaCl, 5; Na2-ATP, 2; MgCl2, 2; EGTA, 10; HEPES, 10; biocytin, 10 and Alexa 488 hydrazide, 0.2 (Invitrogen); pH 7.4. Voltage-clamp recordings were performed using Axopatch 200B (Molecular Devices). Voltage steps were generated with Clampex10 (Molecular Devices) and data analysed with pClamp10 (Molecular Devices). Seal resistances were between 4 and 18 GΩ. In voltage clamp mode, voltage steps were applied from −70 mV holding potential to levels ranging from −100 to 40 mV in 10 mV steps. To subtract leak currents we used a P4 protocol provided by Clampex10 that allowed simultaneous storage of raw and leak subtracted data.

**Biomolecule coupling on gel surface.** Briefly, acrylamide (60 mg, 6%w/v), acrylic acid (6 µL, 0.6% w/v) and N,N-methylene-bis-acrylamide were dissolved in PBS (1 mL), and pH of solution was adjusted to pH 8. Methylsulfone comonomer (N-(4-(5-(methylsulfonyl)-1,3,4-oxadiazol-2-yl)phenyl)acrylamide) (4 mg, 0.4% w/v) was dissolved in DMF (100 µL) and mixed with above solution. The ammonium persulfate (initiator, 10% solution, 1/100 of total volume) and TEMED (catalyst, 1/1000 of total volume) was added to it under nitrogen. One drop of monomer solution (6 µL for 0.2, 10 µL for 2 and 20 kPa) was immediately placed on Sigmacote-coated glass slide and covered with 3-acryloxypropyl-trimethoxysilane functionalized coverslips. For binding PL, the gel was activated by 0.2 M EDC (N-(3-dimethylaminopropyl)-N′- ethylcarbodiimide hydrochloride), 0.1 M NHS (N-hydroxysuccinimide), 2-(N-morpho)-ethanesulfonic acid (0.1 M ) and NaCl (0.5 M) for 15 min, followed by washing and 1 h coupling with (10 µgmL-1) PL solution in PBS. Bioconjugation of CSRARKQAASIKVAVSADR (IKVAV) (100 µgmL-1) was performed by placing a drop (20 µL) of peptide solution in PBS, on the gel surface and coupling for 1 h at r.t. For orthogonal coupling hydrogel was activated with EDC/NHS and incubated with PL solution (10 µgmL-1) for 1 h followed by washing and coupling with IKVAV solution (100 µgmL-1). The IKVAV/PL mixture was covalently bonded on APTES functionalized glass, activated by incubation with aq. glutaraldehyde solution (8%) for 6 h, subsequent washing and incubation with IKVAV/PL (100/10 µgmL-1) solution.

# References

Qin, Y., Capaldo, C., Gumbiner, B.M., and Macara, I.G. (2005). The mammalian Scribble polarity protein regulates epithelial cell adhesion and migration through E-cadherin. The Journal of cell biology *171*, 1061-1071.
